# Potential Strategies for Overcoming Drug Resistance Pathways Using Propolis and Its Polyphenolic/Flavonoid Compounds in Combination with Chemotherapy and Radiotherapy

**DOI:** 10.3390/nu16213741

**Published:** 2024-10-31

**Authors:** Nada Oršolić, Maja Jazvinšćak Jembrek

**Affiliations:** 1 Division of Animal Physiology, Faculty of Science, University of Zagreb, Rooseveltov trg 6, HR-10000 Zagreb, Croatia; 2 Division of Molecular Medicine, Laboratory for Protein Dynamics, Ruđer Bošković Institute, Bijenička cesta 54, HR-10000 Zagreb, Croatia; maja.jazvinscak.jembrek@irb.hr; 3 School of Medicine, Catholic University of Croatia, Ilica 244, HR-10000 Zagreb, Croatia

**Keywords:** cancer, propolis, polyphenolic/flavonoid compounds, chemotherapy, radiotherapy, chemosensitization, radiosensitization, healthy cells protection

## Abstract

Conventional cancer treatments include surgical resection, chemotherapy, hyperthermia, immunotherapy, hormone therapy, and locally targeted therapies such as radiation therapy. Standard cancer therapies often require the use of multiple agents, which can activate nuclear factor kappa B (NF-κB) in tumor cells, leading to reduced cell death and increased drug resistance. Moreover, the use of multiple agents also contributes to added toxicity, resulting in poor treatment outcomes. Cancer cells gradually develop resistance to almost all chemotherapeutics through various mechanisms, such as drug efflux, alterations in drug metabolism and transport, changes in signal transduction pathways, enhanced DNA repair capacity, evasion of apoptosis, increased mutations, reactivation of drug targets, interaction with the cancer microenvironment, cancer cell-stroma interactions, epithelial–mesenchymal transition (EMT)-mediated chemoresistance, epigenetic modifications, metabolic alterations, and the effect of cancer stem cells (CSCs). Developing new strategies to improve chemotherapy sensitivity while minimizing side effects is essential for achieving better therapeutic outcomes and enhancing patients’ quality of life. One promising approach involves combining conventional cancer treatments with propolis and its flavonoids. These natural compounds may enhance tumor response to treatment while reducing toxicity. Propolis and its components can sensitize cancer cells to chemotherapeutic agents, likely by inhibiting NF-κB activation, reprogramming tumor-associated macrophages (TAMs; an M2-like phenotype), and thereby reducing the release of matrix metalloproteinase (MMP)-9, cytokines, chemokines, and the vascular endothelial growth factor (VEGF). By reducing TAMs, propolis and its components may also overcome EMT-mediated chemoresistance, disrupt the crosstalk between macrophages and CSCs, inhibit the maintenance of stemness, and reverse acquired immunosuppression, thus promoting an antitumor response mediated by cytotoxic T-cells. This review highlights the potential of flavonoids to modulate the responsiveness of cancer to conventional treatment modalities. The evidence suggests that novel therapeutic strategies incorporating flavonoids could be developed to improve treatment outcomes. The positive effects of combining propolis with chemotherapeutics include reduced cytotoxicity to peripheral blood leukocytes, liver, and kidney cells. Therefore, polyphenolic/flavonoid components may hold potential for use in combination with chemotherapeutic agents in the clinical treatment of various types of cancers.

## 1. Introduction

Cancer-related deaths have increased by 40% in recent years [1], and it is projected that cancer cases could increase by 60% over the next 20 years [2]. According to the most recent Global Cancer Report from the World Health Organization/International Agency for Research on Cancer (WHO/IARC), approximately 19.3 million new cancer cases and about 10 million cancer-related deaths occurred worldwide in 2020. In the United States alone, an estimated 1,958,310 new cancer cases were expected in 2023, equating to more than 5000 new cases per day. The number of cancer patients is anticipated to rise even further, with up to 13 million cancer-related deaths projected by 2030 [2]. In low-income regions, such as Africa, cancer cases are expected to increase by 70% by 2030 due to population growth, aging, and the emergence of new infectious diseases like malaria, Ebola, AIDS, and COVID-19 [3].

Aggarwal et al. [4] attributed nearly 90–95% of all cancers to lifestyle factors, including exposure to environmental toxins such as tobacco smoke, asbestos, styrene, formaldehyde, and tetrachloroethylene, as well as infections, stress, obesity, diet, physical inactivity, and alcohol consumption. Only 5–10% of cancers are associated with genetic reasons. Consequently, around 25–30% of cancer-related deaths are due to tobacco use, 30–35% are linked to diet, 15–20% are due to infections, and the remaining percentage is attributed to other factors like radiation, stress, physical inactivity, and environmental pollutants.

Traditional cancer treatments include surgery, radiotherapy, chemotherapy, and immunotherapy, either alone or in combination. However, these methods are often rigorous and can cause significant side effects, damage to other tissues and organs, and the development of resistance. Chemotherapy, while commonly used in both early and late stages of cancer, is associated with side effects such as neutropenia, myalgia, nausea, fatigue, vomiting, diarrhea, alopecia, and neuropathy, which can severely impact patients’ quality of life.

Natural compounds, including propolis and its flavonoids, have shown significant pharmacological potential as complementary or alternative therapies for the prevention and treatment of various cancers. These compounds may offer strategies to overcome drug resistance or serve as chemosensitizers in adjunct cancer therapy. Studies, including our own, have shown that propolis exhibits strong cytostatic, anti-carcinogenic, and antitumor effects in both in vitro and in vivo tumor models [5]. The therapeutic activities of propolis are believed to be largely dependent on the presence of flavonoids, which have been reported to stimulate the immune system, scavenge oxygen radicals, and regulate numerous tumor-related processes, such as oxidative stress, cell proliferation, cell cycle, apoptosis, angiogenesis, and metastasis by modulating various cell signaling pathways. Scientific evidence and observational studies suggest that flavonoids can target specific molecular signaling pathways, including p53, epidermal growth factor receptor (EGFR), VEGF, apoptosis, phosphatidyl inositol-3-kinase (P13K)/Akt/mammalian target of rapamycin (mTOR), extracellular signal-regulated kinase 1/2 (ERK1/2) that belongs to a subfamily of mitogen-activated protein kinases (MAPKs), signal transducer and activator of transcription (STAT), NF-κB, and Wnt/β-catenin pathways [1,2,3,4,5,6,7,8,9].

In our previous paper [5], we listed numerous mechanisms through which propolis and its flavonoids exert chemopreventive effects and inhibit tumor growth. Propolis and its compounds exhibit anticancer effects through multiple mechanisms, such as halting the cell cycle, inhibiting cancer cell growth, reducing CSCs, and triggering apoptosis. They also influence oncogenic signaling pathways, inhibit MMPs, prevent metastasis, and suppress angiogenesis and inflammation. Additionally, propolis modulates the tumor microenvironment, influences macrophage activity, regulates epigenetic changes, and has antiviral and antibacterial properties. It also plays a role in controlling glucose metabolism and modulating gut microbiota. The major active components of propolis include caffeic acid phenethyl ester (CAPE), galangin, chrysin, acacetin, fisetin, nemorosone, propolin G, artepillin C, cardanol, cardol, pinocembrin, pinobanksin, chicoric acid, and two types of phenolic acids such as hydroxycinnamic acids (caffeic acid, ferulic acid, cinnamic and coumaric acid) and hydroxybenzoic acids (salicylic, gallic, protocatechinic, vanillic and gentisic acid) as well as luteolin, apigenin, myricetin, naringenin, kaempferol, quercetin, polysaccharide, tannins, terpenes, sterols and aldehydes [5,6,7,8,9].

When combined with various forms of cancer therapy (such as chemotherapy, radiotherapy, and hyperthermia), these processes can significantly contribute to overcoming chemo- and radioresistance. In this paper, we will explore the mechanisms by which propolis and its components enhance sensitization to chemotherapy and radiotherapy, with a focus on the positive and negative consequences on both tumor and healthy cells. It is well known that standard cancer therapy can cause acute toxicity, which can be severe enough to necessitate discontinuation of treatment. Agents that can reduce the toxicity of standard therapies on normal cells, or that can enhance the response of tumor cells to these therapies, have the potential to significantly improve the current management of cancer. Additionally, maintaining normal hematopoiesis in cancer patients is critical for the success of any treatment. Anemia, the most common hematological manifestation of cancer, affects 40–64% of patients undergoing treatment for malignancies, and its incidence increases with the administration of chemotherapy or radiotherapy [10]. According to Gaspar et al. [10], anemia in cancer can be broadly classified into four major categories: hypoproliferative anemia, hemolytic anemia, anemia of miscellaneous etiology, and anemia of uncertain etiology.

Anemia in cancer patients can result from bleeding, either on the surface of tumor or internally, as well as from decreased production and shorter lifespan of erythrocytes [11]. While hemoglobin levels may remain normal, the immune functions associated with red blood cells are important for effective tumor treatment. Erythrocytes perform functions such as enhancing phagocytosis, identifying and carrying antigens, eliminating immune complexes from circulation, and strengthening T-cell-dependent reactions [12,13,14]. Anemia directly impairs the immune functions of erythrocytes, thus negatively affecting tumor treatment.

**Figure 1:**
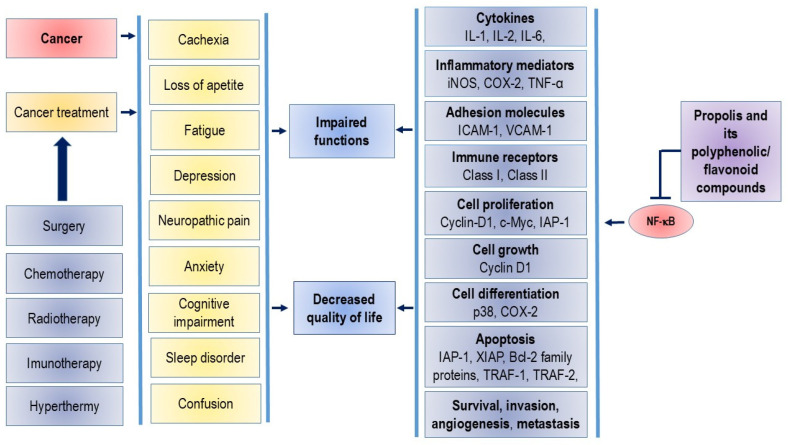
A schematic illustration depicting the effects of cancer and various treatments on patient outcomes, as well as the potential role of propolis and its polyphenolic compounds in modifying NF-kB signaling. Propolis and its components influence several key processes, including inflammation, cell differentiation, proliferation, tumor growth, invasion, angiogenesis, metastasis, and overall survival. NF-κB activation triggers the expression of pro-inflammatory cytokines and genes encoding proteins like IAP-1, XIAP, and Bcl-2, which are involved in adaptive stress responses and are crucial for tumor cell development by affecting multiple steps in these signaling pathways. Abbreviations: NF-κB, nuclear factor kappa-light-chain-enhancer of activated B cells; IL-1, interleukin-1; IL-2, iterleukin-2; IL-6, interleukin-6; COX-2, cyclooxygenase-2; TNF-α, tumor necrosis factor alpha; iNOS, inducible nitric oxide synthase; ICAM-1, Intercellular Adhesion Molecule 1; VCAM-1, vascular cell adhesion protein 1, c-Myc, proto-oncogene protein; IAP, inhibitors of apoptosis proteins; XIAP, X-linked inhibitor of apoptosis protein; Bcl-2, B-cell lymphoma 2; TRAF-1, TNF receptor-associated factor 1; TRAF-2, TNF receptor-associated factor 2; p38, p38 kinase.

Many studies suggest that preparations of propolis and related flavonoids may enhance the anticancer efficacy of chemotherapeutic agents while reducing their cytotoxicity to immunocompetent cells [13,14,15,16,17,18,19,20]. Pretreatment with flavonoids, both in vitro and in vivo, sensitizes cancer cells to the growth inhibition and apoptosis induced by chemotherapeutic agents. This is likely due to the ability of flavonoids to inhibit P-glycoprotein (P-gp) activity, which increases the accumulation of chemotherapeutics in P-gp-expressing cancer cells. P-gp, also known as multidrug resistance protein 1 (MDR1), ATP-binding cassette sub-family B member 1 (ABCB1), or cluster of differentiation 243 (CD243), is a key membrane protein that acts as an ATP-dependent efflux pump. Inhibition of P-gp activity leads to altered absorption and bioavailability of chemotherapeutic drugs when co-administered with flavonoids [21,22,23,24]. As a result, polyphenolic and flavonoid components may serve as potent adjuncts to various cancer therapies, including surgery, chemotherapy, radiotherapy, immunotherapy, and hyperthermic intraperitoneal chemotherapy (HIPEC) (Figure 1).

Phenolic compounds from propolis also exhibit synergistic effects with cytotoxic drugs, enhancing their medicinal benefits. In general, synergism occurs when the interaction between two or more substances produces an effect greater than the sum of their individual effects. Synergy can be either additive or reversal, with additive synergy occurring when the interaction effect is enhanced. Phenolic compounds, such as chrysin, apigenin, ferulic acid, and quercetin, have been shown to produce synergistic effects when combined with medically approved drugs, such as 5-fluorouracil, tamoxifen, and doxorubicin, against various cell lines originating from breast cancer [13,14,15,16,17,18,19,20,25,26,27,28,29,30,31,32].

In addition, numerous studies have demonstrated that propolis and its flavonoids enhance the antitumor effects of chemotherapeutic agents in both in vitro and in vivo models by inhibiting cellular proliferation through the suppression of NF-κB activation and other genetic targets within the NF-κB signaling cascade [33,34,35]. For example, in vivo studies using nude mice injected with pancreatic cancer cells and treated with both curcumin and gemcitabine showed a significant reduction in tumor volume, Ki-67 proliferation index, NF-κB activation, and the expression of NF-κB gene products (such as cyclin D1, c-Myc, B-cell lymphoma-2 (Bcl-2), Bcl extra large (Bcl-xL), cellular inhibitor of apoptosis protein 1 (CIAP-1), cyclooxygenase-2 (COX-2), MMPs, and VEGF). These studies also observed suppression of angiogenesis compared to control animals [34,35].

NF-κB plays a crucial role in the development and progression of cancer, as it regulates over 400 genes involved in inflammation, cell survival, proliferation, invasion, angiogenesis, and metastasis [36]. Many chemotherapy drugs, such as platinum-based agents, anthracyclines, and taxanes, are known to promote the activation of the NF-κB pathway [37]. The presence of constitutively active NF-κB is a common underlying factor in inflammation-related cancers, and it leads to the constitutive expression of various prosurvival genes, including (i) anti-apoptotic genes like Bcl-2 and Bcl-xL; (ii) proangiogenic gene VEGF; and (iii) genes that encode proteins involved in metastasis and invasion, such as MMPs. Consistent with this, both clinical and epidemiological data suggest that NF-κB inhibitors have strong chemopreventive potential. Chemoprevention refers to the use of chemicals, including food supplements, to prevent the development and progression of cancer. Numerous studies have shown that propolis and flavonoids act as chemopreventive agents by suppressing NF-κB-mediated inflammation and cancer. Due to their multifunctional properties, propolis and its polyphenolic compounds are promising agents in cancer treatment, capable of blocking NF-κB activation, inducing apoptosis, and inhibiting proliferation, invasion, metastasis, and angiogenesis.

Constitutive NF-κB activity has been detected in numerous human cancers, such as breast, non-small cell lung, thyroid, T- and B-cell leukemia, melanoma, colon, bladder, and various virus-associated tumors [5,9,17,37,38,39,40,41,42,43].

Blocking NF-κB has been shown to reduce tumor growth in these cancers. Genetic alterations in NF-κB-related genes further support its involvement in cancer development. While inhibiting NF-κB often triggers apoptosis in cancer cells [5,44,45], in some cases, its activation actually prevents tumor growth. This indicates that NF-κB’s role in cell regulation varies by tissue type. Targeting NF-κB remains a key therapeutic strategy for cancer, inflammation, and autoimmune diseases (Figure 1). Furthermore, flavonoids, whether present in propolis or consumed through the diet, can attenuate or inhibit the initiation, promotion, and progression of cancer by interacting with various enzymes and receptors in pathways that regulate cell proliferation, differentiation, apoptosis, inflammation, angiogenesis, metastasis, and reversal of multidrug resistance. Flavonoids can also reduce DNA damage and prevent mutations, thereby increasing genomic stability and decreasing cancer risk. Their selective nature makes them suitable for protecting normal cells while inducing cell death in cancer cells during chemotherapy or radiotherapy. Additionally, a better understanding of epigenetic processes can provide a basis for the improvement of their clinical efficacy.

## 2. Mechanisms of Tumor Cell Chemoresistance

Chemotherapy is a cornerstone in cancer treatment, often combined with surgery, radiation, or biological therapies. However, chemotherapeutic agents are nonselective, targeting both healthy and tumor cells that are actively dividing. The primary goal of chemotherapy is to eliminate rapidly dividing tumor cells, but differences in cell cycle entry, membrane properties, repair mechanisms, antioxidant capacity, and vascular systems between tumor and normal cells result in varying levels of toxicity and slower recovery in cancer cells. Over time, cancer cells develop resistance, either primary or acquired, to nearly all chemotherapeutic agents through various mechanisms. Approximately 90% of cancer progression during and after chemotherapy is linked to drug resistance [46], with prolonged exposure to chemotherapy often leading to a phenomenon known as multidrug resistance (MDR). MDR can result from several mechanisms, including (i) ATP-binding cassette (ABC) transporters that pump out chemotherapeutics, thereby reducing their efficacy; (ii) oncogene mutations that can render cancer cells resistant to previously effective treatments; (iii) adaptation of cancer cells to the microenvironment; (iv) surviving CSCs that can evade conventional therapies; and (v) activation of growth factors and cell defense mechanisms.

**Figure 2:**
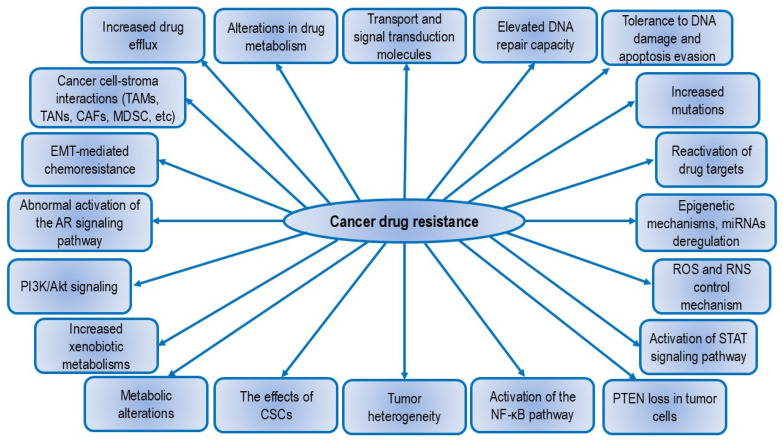
Key mechanisms of drug resistance in cancer against chemotherapy and targeted therapies. Resistance arises due to a variety of factors, including individual genetic differences, mutations, altered epigenetics, target gene amplification, enhanced DNA repair, increased drug efflux, reduced drug uptake, and inhibition of cell death mechanisms. Additionally, resistance against anticancer agents can result from cellular senescence (suppression of apoptosis), alterations of drug metabolism and drug targets, and molecular and cellular changes, such as altered activity or expression of surface receptors, transporters, and drug targets, including P-gp, MRP1, Topo, p53, and breast cancer gene 1 (BRCA1). Drug resistance can also depend on cellular autophagy and hypoxic conditions. Abbreviations: AR, androgen receptor; TAMs, tumor-associated macrophages; TANs, tumor-associated neutrophils; MDSCs, myeloid-derived suppressor cells; PTEN, phosphatase and tensin homolog; NF-κB, nuclear factor kappa-light-chain-enhancer of activated B cells; CSCs, cancer stem cells; ROS, reactive oxygen species; RNS, reactive nitrogen species; STAT, signal transducer and activator of transcription.

The ABC transporters superfamily plays a prominent role in chemoresistance to various anticancer drugs, particularly through proteins such as multidrug resistance-associated protein 1 (MRP1)/MRP2, P-gp, and breast cancer resistance protein (BCRP). Mechanisms involved in cancer drug resistance include (i) increased drug efflux, (ii) alterations in drug metabolism, (iii) changes in transport and signal transduction molecules, (iv) enhanced DNA repair capacity and tolerance to DNA damage, (v) evasion of apoptosis, (vi) increased mutations, (vii) reactivation of drug targets, (viii) interaction with the tumor microenvironment and cancer cell-stroma interactions, (ix) EMT-mediated chemoresistance, (x) genetic factors such as abnormal activation of the androgen receptor (AR) signaling pathway, (xi) epigenetic mechanisms and dysregulation of miRNAs, (xii) alterations of the PI3K/Akt signaling, (xiii) increased xenobiotic metabolism and metabolic changes, (xiv) the effects of CSCs, (xv) tumor heterogeneity, (xvi) reactive oxygen species (ROS) and reactive nitrogen species (RNS) control mechanism, (xvii) activation of the STAT signaling pathway, (xviii) activation of the NF-κB pathway, and (xix) loss of phosphatase and tensin homolog (PTEN) in tumor cells [15,47,48,49]. Additional resistance mechanisms include the overexpression of aldehyde dehydrogenase (ALDH), endoplasmic reticulum (ER) stress, and involvement of the receptor for advanced glycation end products (RAGE), NF-kB, and galectin-3. TAMs and tumor-associated neutrophils (TANs) also contribute to chemo- and radioresistance, and their removal may enhance the effectiveness of tumor treatments [5,15]. According to Labialle et al. [50], several genes and signaling pathways regulate P-gp activity and expression, including p53, early growth response protein-1 (EGR1), Ras, Raf, retinoic acid receptor (RAR) α/β, c-fos, c-jun, protein kinase C (PKC), protein kinase A (PKA), and NF-κB (Figure 2).

Several studies suggest that propolis and its flavonoids, when used in combination with chemotherapeutic agents, can affect all these mechanisms, making them a promising strategy to overcome chemotherapeutic resistance and MDR in tumors [15,16,17].

## 3. Propolis and Its Polyphenolic/Flavonoid Compounds in Combination with Chemotherapy

### 3.1. Reducing Toxicity, Enhancing Chemosensitivity, and Overcoming Resistance with Propolis and Its Polyphenolic/Flavonoid Compounds

Numerous studies have shown that various propolis preparations, regardless of geographical origin, can enhance the efficacy of anticancer drugs while reducing their side effects and drug resistance through multiple mechanisms.

The beneficial effects of propolis and flavonoids in combination with chemotherapy are well-documented [15,16,17,18,19,25,26,27,28,29,30,31,32,33,34,35]. Propolis, considered non-toxic and without side effects, has been shown to reduce the toxic effects of several chemotherapeutics, including vincristine, vinblastine, tamoxifen, paclitaxel, docetaxel, 5-fluorouracil (5-FU) doxorubicin, methotrexate, gentamicin, cisplatin, temozolomide, irinotecan, cerulean (nanopharmaceuticals), as well as drugs used for cancer pain management like acetaminophen and paracetamol. Some studies suggested that combining propolis or specific flavonoids with epirubicin, 5-FU, doxorubicin, cisplatin, irinotecan, and vincristine may represent a new approach for cancer treatment, potentially enhancing the host immune response and lowering chemotherapy doses to reduce side effects [15,16,17,18,19].

#### 3.1.1. Effectiveness of Propolis in Interactions with Antimetabolites

Antimetabolites are anticancer agents structurally similar to natural substrates but possess enough differences to disrupt their metabolism. This group includes folic acid antagonists, purine antimetabolites, and pyrimidine antimetabolites. Antimetabolites interfere with nucleic acid synthesis by disrupting the production of key nucleotide metabolites or replacing natural metabolites. While they are cytotoxic to cancer cells, they also exhibit significant toxicity to normal cells, particularly those in the bone marrow and gastrointestinal system.

##### 5-FU

5-FU, a pyrimidine analog, disrupts nucleoside metabolism by incorporating into RNA and DNA, leading to cytotoxicity. In mammalian cells, it is converted to FdUMP, which inhibits thymidylate synthase (TS) and disrupts DNA replication. Resistance to 5-FU arises from the overexpression of metabolic enzymes like TS, dihydropyrimidine dehydrogenase (DPD, which inactivates 5-FU), and thymidine phosphorylase (TP) [51]. TS overexpression also enhances the activity of deoxyuridine triphosphatase, methylation of the MutL protein homolog 1 (MLH1) gene, and anti-apoptotic pathways (e.g., Bcl-2, Bcl-xL, and Mcl-1 proteins) [51,52,53,54,55]. Resistance can be countered by inhibiting these proteins and XIAP expression, a direct inhibitor caspase 3, 7, and 9), and promoting pro-apoptotic factors such as Bax and Bcl2-associated agonist of cell death (Bad), as well as targeting CSCs markers (NOTCH1, CD44, ALDHA1, Oct4, SOX2, and Nanog), autophagy, EMT, miRNA, epigenetics, and redox balance [51,52,53,54,55].

The inducible activation of the nuclear NF-κB has been identified as a key inhibitor of the apoptotic response to chemotherapy and radiation [52]. Studies have shown that flavonoid quercetin (100 µM), when combined with 5-FU (0.1 or 0.2 µM), significantly inhibits growth and promotes apoptosis in EC9706 and Eca109 esophageal cancer cells compared to either quercetin or 5-FU alone. These effects are associated with decreased expression of phosphorylated IκBα (pIκBα), a molecule activated by 5-FU exposure. Similarly, curcumin in combination with either 5-FU or 5-FU + oxaliplatin (FOLFOX), has shown significantly greater growth inhibition and increased apoptosis in HCT116 and HT29 colon cancer cells than any treatment alone (curcumin, 5-FU, curcumin + 5-FU, or FOLFOX) [53]. These effects were linked to reduced expression and activation of EGFR, HER-2, HER-3, and insulin-like growth factor 1 receptor (IGF-1R), along with their downstream signaling targets such as Akt and COX-2 [54].

Like quercetin, kaempferol (15–120 µM), when combined with 5-FU (60 µM), exerts a synergistic effect by inhibiting cell viability, promoting apoptosis, and inducing cell cycle arrest in both chemoresistant and sensitive colon cancer LS174 cells. The 5-FU-resistant LS174-R cells are characterized by the upregulation of ABC subfamily G member 2 (ABCG2) and MDR1. Kaempferol also reduces ROS production and modulates key signaling pathways, including JAK/STAT3, MAPK, PI3K/AKT, and NF-κB. Additionally, kaempferol increases the proportion of apoptotic cells by approximately 40% in 5-FU-sensitive cells and 25% in 5-FU-resistant cells. In leukemia HL-60 and NB4 cells, kaempferol has also demonstrated the ability to inhibit MDR by downregulating ABCB1, ABCC1, Akt, and Bcl2 [55].

Several studies highlight flavonoids as important modulators or substrates of intestinal ABC transport proteins [56,57,58,59]. The inhibition of ABC transporters by flavonoids may affect the bioavailability of drugs, bioactive food compounds, and food-borne toxins following oral ingestion. This flavonoid-mediated interaction with intestinal ABC transport proteins may explain unexpected food–drug, food–toxin, or food–food interactions.

Combining drug treatment with natural antioxidants as adjuncts to standard cancer therapies could be a promising strategy to overcome resistance. Many researchers suggest that antioxidants in combination with chemotherapy can enhance the efficacy of antineoplastic drugs while minimizing their adverse effects on normal tissues [13,14,15,16,17,18,19,56,57,58,59]. A review of different flavonoids and their effects as ABC transporter inhibitors on MDR, intracellular accumulation, and bioavailability of bioactive compounds is provided by Li et al. [58] and Michalak and Wesolowska [59]. Flavonoids from different groups, including flavones (chrysin and apigenin), flavonols (kaempferol), isoflavones (genistein and biochanin A), and flavanones (naringenin), are emerging as potent inhibitors of ABCB1 and ABCG2, with effects comparable to known inhibitors such as verapamil and cyclosporine. Some flavonoids, like quercetin, myricetin, kaempferol, and apigenin, also act as chemosensitizers by inhibiting ATPase activity of ABC transporters, thereby achieving a synergistic effect with chemotherapeutic agents in promoting programmed cell death in tumors [58,59].

Toden et al. [60] investigated the chemosensitizing effects of curcumin in combination with 5-FU and found that the combination of these two compounds inhibits cell proliferation and induces apoptosis in both parental and 5-FU-resistant cells. This treatment also suppressed EMT by downregulating polycomb repressive complex subunit (a catalytic multi-subunit complex involved in transcriptional repression through the methylation of lysine 27 at histone 3) inducers (BMI1, SUZ12, and EZH2).

Propolis, when used in combination with 5-FU, has shown potential in reducing the formation of various cancers, including colorectal, breast, rectal, ovarian, bladder, and liver cancers. This effect is primarily achieved by inhibiting the expression of proinflammatory cytokines (tumor necrosis factor alpha (TNF-α) and interleukin (IL)-2), COX-2, inducible nitric oxide synthase (iNOS) and β-catenin proteins [61]. In a mouse model of colorectal cancer, the combined use of propolis ethanol extract at the doses of 10, 30, or 90 mg/kg (5 times a week) and 5-FU at 50 mg/kg (once a week during a period of 8 weeks) reduced the number of aberrant crypt foci and pathological lesions compared to either the control group or 5-FU alone [61]. The anti-inflammatory activity of this combination, through the reduction of COX-2, iNOS, and β-catenin expression, likely underlies this effect [61].

Propolis also enhances the anti-tumor effect of 5-FU by increasing the lymphocyte/polymorphonuclear leukocyte ratio and reducing toxic side effects such as impaired erythropoiesis, anemia, loss of appetite and taste, diarrhea, and oral mucositis. Suzuki et al. [62] demonstrated that oral administration of crude water-soluble propolis (CWSP, 13 mg/kg/day) together with 5-FU (50 mg/kg/day) or mitomycin (MMC, 1 mg/kg/day for 35 days), significantly increased tumor regression compared to chemotherapy alone. The combination of CWSP with 5-FU or MMC also mitigated chemotherapy-induced cytopenia, helping in the recovery of white and red blood cells [62]. However, Sobocanec et al. [63] found that propolis in combination with 5-FU had a gender-specific impact on tumor growth and metastasis, showing more favorable effects in males, possibly due to decreased dihydropyrimidine dehydrogenase (DPD) protein level, which increased 5-FU sensitivity and prolonged its effects.

The observed combined antitumor effects of native propolis and 5-FU may be attributed to polyphenols/flavonoids and other phytochemicals in propolis. Compounds like quercetin, curcumin, resveratrol, EGCG, luteolin, sulforaphane, berberine, genistein, and capsaicin have been shown to participate in cancer prevention and therapy, particularly concerning CSCs [64,65,66,67]. Chrysin, galangin [68,69], caffeic acid, and naringenin [70,71,72,73] also show anticancer activities. In Croatian propolis, the most abundant compounds with anti-tumor activity were chrysin and galangin, followed by kaempferol, naringenin, and caffeic acid, while quercetin concentration was relatively low. It seems that the biological properties of propolis result from the combined effects of all its components, with no single constituent exhibiting activity greater than the total extract [5,8,63].

The antitumor efficacy of propolis may be enhanced by its ability to prevent TAM from polarizing into the M2 phenotype, instead promoting the M1 phenotype, which supports immunogenic cell death via the TNF/TNF-related apoptosis-inducing ligand (TRAIL) pathway [74]. Additionally, propolis could inhibit the STAT3/hypoxia-inducible factor (HIF)-1 signaling pathway [75], which is critical in the hypoxic response and M2 polarization of TAMs [5,76]. Under hypoxic conditions, HIF-1 signaling is activated, resulting in the altered expression of its target genes, such as VEGF and IL-6. Thereby, by reversing this polarization and regulating the tumor immune microenvironment, propolis, in combination with 5-FU, may offer a promising approach to overcoming chemoresistance.

Moreover, 5-FU is associated with a range of severe side effects, including cardiotoxicity and cardiovascular diseases. 5-FU-induced cardiotoxicity is the second cause of cardiotoxicity caused by chemotherapeutic agents. This cardiotoxicity is dose-dependent. Factors such as type of drug, comorbidities, and chemotherapy regimen may influence its incidence, which can vary from 0% to 20% [77]. Notably, 5-FU at lower doses is more toxic to the myocardium than capecitabine. 5-FU cardiotoxicity is linked to several changes in erythrocytes: (i) partial irreversible echinocytosis; (ii) increased membrane fluidity, reducing blood viscosity during infusion; (iii) K+ efflux, possibly causing arrhythmias; (iv) decreased O_2_ and increased 2,3-bisphosphoglycerate (2,3-BPG) levels, lowering hemoglobin’s O_2_ affinity; and (v) a significant drop in ATP levels, leading to altered cell morphology and ion balance [78].

Barary et al. [76] demonstrated that 5-FU induces cardiotoxicity by an increase in malondialdehyde (MDA) levels, cyclooxygenase-2 (COX-2), and tumor necrosis factor-α (TNF-α) expression, cardiac enzyme levels, and histopathological degenerations. 5-FU treatment also decreased body weight, total antioxidant capacity (TAC), catalase levels, blood cell counts, and hemoglobin (Hb) levels. In addition, 5-FU disrupted ECG parameters, including increased elevation in the ST-segment and increased QRS complex and QTc duration. However, treatment with propolis attenuated 5-FU-induced cardiotoxicity in rats. The treatment alleviated body weight loss by reducing oxidative stress, lowering cardiac enzyme levels, preventing histopathological degenerations, decreasing COX-2 expression in cardiac tissue, improving ECG disturbances, and increasing blood cell counts and TAC. In addition to propolis, polyphenols such as resveratrol, quercetin, catechins, naringin, baicalein, genistein, and apigenin have also shown cardioprotective effects [79].

##### Gemcitabine

In a study by Laaroussi et al. [80], propolis reduced oxidative stress induced by gemcitabine, significantly lowering hepatic and renal oxidative stress markers, including catalase and MDA. Additionally, quercetin at various concentrations (6.25, 12.5, 25, and 50 mM) promoted cell death and enhanced gemcitabine (500 nM) sensitivity in human pancreatic cancer MIA Paca-2 and MIA Paca-2 (GEM-resistant) cells by targeting the receptor for advanced glycation end products (RAGE)/PI3K/AKT/mTOR axis, particularly through RAGE inhibition. Flavokawain-B, a chalcone, showed potent anti-cancer effects in gemcitabine-resistant non-small-cell lung cancer (NSCLC) cells by inducing apoptosis and ROS production while inhibiting the PI3K/Akt signaling pathway [81]. Furthermore, Zhou et al. [82] showed that chrysin enhanced the sensitivity of pancreatic cancer cells to gemcitabine by inducing ferroptotic death both in vitro and in vivo. Chrysin directly binds to carbonyl reductase 1 (CBR1), inhibiting its enzymatic activity, which in turn increases cellular ROS levels, leading to ROS-dependent autophagy [82]. The autophagy results in the degradation of ferritin-heavy polypeptide 1 (FTH1) and an increase in the intracellular free iron levels, thereby promoting ferroptosis in pancreatic cancer cells. Thongpon et al. [83] showed that curcumin synergistically enhances the efficacy of gemcitabine against gemcitabine-resistant cholangiocarcinoma (CCA cells, KKU-213B^GemR^) by induction of apoptosis and cell cycle arrest in the S and G2/M phases, partly via inhibiting LAT2/glutamine pathway. Moreover, in vivo cotreatment with curcumin and gemcitabine significantly reduced tumor size and growth rate, as well as LAT2 expression in a gemcitabine-resistant CCA xenograft mouse model. The authors believe that this combination could be an alternative strategy for the treatment of gemcitabine-resistant patients with cholangiocarcinoma (CCA).

#### 3.1.2. Effectiveness of Propolis in Interactions with Alkylating Agents

Alkylating agents are chemotherapy drugs that work by transferring one or more alkyl groups to biological molecules. They damage the DNA by forming bridges (adducts) between DNA strands. These highly reactive compounds can form covalent bonds by particularly targeting DNA at the N-7 position of guanine or, less frequently, at the O-6 position of guanine. The binding results in cross-linking of DNA strands, leading to mutations. Alkylating agents are especially effective against rapidly dividing cells; the DNA damage they cause is often irreversible, resulting in cell death through apoptosis. Examples of alkylating agents are platinum-based drugs such as cisplatin, carboplatin, and oxaliplatin; triazene compounds like dacarbazine and temozolomide; nitrogen blisters (e.g., cyclophosphamide, chlorambucil, melphalan); alkylsulfonates (e.g., busulfan), nitrosourea derivatives (e.g., carmustine, lomustine), and ethyleneimines (e.g., triethylenemelamine, hexamethylmelamine, thiophosphoramide).

##### Cisplatin

Cisplatin is a chemotherapy drug used to treat various cancers, including testicular, ovarian, breast, bladder, head and neck, lung, esophageal, and cervical cancer, mesothelioma, brain tumors, and neuroblastoma. Its mechanism of action involves cross-linking with purine bases on DNA, which interferes with DNA repair, causing DNA damage and inducing apoptosis in cancer cells.

Despite its effectiveness, cisplatin is associated with numerous side effects, including bone marrow suppression, hearing problems (ototoxicity), kidney damage (nephrotoxicity), cardiotoxicity, gastrointestinal disorders, immunosuppression, and other toxicities such as electrolyte imbalance, allergic reactions, and hemorrhage. The toxicity of cisplatin is mediated by several mechanisms, including redox imbalance caused by increased reactive oxygen species production and lipid peroxidation, inflammation, and activation of p53 and its downstream apoptotic pathways [13,14,19].

Several studies have explored combinations of cisplatin with natural compounds to enhance its efficacy and reduce resistance. For example, a combination of morin (a known inhibitor of NF-κB) and cisplatin was found to synergistically sensitize cisplatin-resistant ovarian cancer SK-OV-3 cells by downregulating galectin-3 [84]. Galectin-3 plays a crucial role in cell–cell adhesion, cell–matrix interactions, macrophage activation, angiogenesis, metastasis, and apoptosis. Additionally, morin hydrate was shown to reverse resistance in cisplatin-resistant hepatocellular cancer HepG2^DR^ cells by impairing poly (ADP-ribose) polymerase (PARP-1)/ high mobility group box 1 (HMGB1)-dependent autophagy [85]. Pre-treatment with quercetin was found to resensitize SKOV-3/cisplatin cells to cisplatin by activating the mitochondrial apoptotic pathway (via cleavage of PARP and caspases 9, 7, and 3) and downregulating the mTOR/STAT3 signaling pathway [86].

Hesperetin has also been shown to sensitize cisplatin-resistant human lung cancer cells (A549/DDP) to cisplatin during in vivo and in vitro studies by reducing P-gp expression and increasing intracellular accumulation of P-gp substrates like rhodamine 123 [87]. Similarly, Zhao et al. [88] demonstrated that poncirin (2.5 to 80 μM), a flavanone glycoside, increased sensitivity to cisplatin (8 μM) in cisplatin-resistant osteosarcoma cells by downregulating the expression of MDR-1, MRP1 (ABCC1), and BCRP, and inhibiting the PI3K/Akt signaling pathway. Moreover, fisetin was found to enhance the sensitivity of chemoresistant head and neck carcinoma cells (Cal27/DDP and FaDu/DDP) to cisplatin, possibly through the heat-shock protein 90 alpha family class A member (heat-shock protein (HSP)90AA1)/IL-17 pathway [89]. Apigenin was reported to sensitize human CD44+ prostate cancer stem cells to cisplatin therapy by enhancing the cytotoxic and apoptotic effects of cisplatin through Bcl-2 regulation. Furthermore, this combination inhibits PI3K and Akt phosphorylation and NF-κB protein expression, acting on the cell cycle by regulating p21, as well as cyclin-dependent kinases CDK-2, -4, and -6. The inhibitory effect of apigenin in combination with cisplatin is manifested in the inhibition of cell migration through the downregulation of snail expression [64].

Other polyphenols, such as quercetin, luteolin, fisetin, caffeic acid phenethyl ester (CAPE), and curcumin, have also demonstrated anticancer, chemosensitizing, and cytoprotective effects against cisplatin-induced toxicity in normal cells. For example, CAPE has been shown to enhance the sensitivity of gastric cancer cells to doxorubicin and cisplatin by inhibiting proteasome function, while Li et al. demonstrated that quercetin acts as an anticancer drug by suppressing cancer aggressiveness and cisplatin resistance in nasopharyngeal carcinoma through modulation of the Yes-associated protein/Hippo signaling pathway [90].

The ability of flavonoids to enhance the antitumor activity of cisplatin by inducing apoptosis and cell cycle arrest may be partially mediated through epigenetic regulation. Thus, flavonoids such as apigenin, quercetin, chrysin, naringenin, daidzein, kaempferol, and myricetin have potentiated the antitumor effects of cisplatin in NSCLC cells in vitro by inhibiting histone deacetylases (HDAC). Among these, apigenin was found to be the most effective HDAC inhibitor. The combination of cisplatin and apigenin resulted in significantly greater S phase prolongation, G2/M cell cycle arrest, and apoptosis compared to either treatment alone, largely due to the induction of p21 and p53 upregulated modulator of apoptosis (PUMA) expression [91]. These effects were more pronounced in p53-proficient NSCLC cells than in p53-null cells. Mechanistically, apigenin was found to reduce HDAC1 binding while increasing the recruitment of RNA polymerase II and Sp1 to p21 and PUMA promoters [92].

Studies by Oršolić et al. [8,19,93] have demonstrated that water and ethanolic preparations of propolis (WSDP, EEP, 50 mg/kg) can increase the therapeutic effects of cisplatin (10 mg/kg) and/or doxorubicin (20 mg/kg) when used in combination in mice. Propolis preparations administered preventively and/or therapeutically can increase the lifespan of treated mice, reduce tumor burden, and decrease the side effects of chemotherapy. Intraperitoneal administration of propolis preparations along with cisplatin inhibited tumor growth when given either before tumor cell inoculation or after tumor growth stabilization. Mice treated with propolis and cisplatin before tumor cell inoculation had a significantly prolonged lifespan compared to control (increase in lifespan, ILS: WSDP + CIS = 100%; EEP + CIS = 39,42%). In mice treated curatively, the ILS for both groups (WSDP + CIS, EEP +CIS) was around 36%. Notably, EEP, in combination with doxorubicin, was more effective in curatively treated mice than in preventively treated ones, consistent with results observed in metastasis formation. Moreover, propolis preparations significantly reduced the toxicity of chemotherapy, as indicated by the maximum percentage of body weight loss. Most in vivo and in vitro studies suggest that certain antioxidants, when used under carefully controlled conditions, can significantly reduce the toxicity of chemotherapeutic agents without compromising, and in some cases even enhancing, their antitumor effects [29,94,95,96,97,98,99,100]. Many compounds that mitigate cisplatin-induced nephrotoxicity contain a nucleophilic sulfur atom, which can bind to the platinum ion, neutralizing its cytotoxic effects without reducing its effectiveness [99,100].

Approximately 30–50% of patients develop acute kidney injury (AKI) following cisplatin treatment, primarily due to apoptosis and necrosis of renal tubule cells [101]. The nephrotoxic effect of cisplatin is both cumulative and dose-dependent, often requiring dose reduction or discontinuation due to excessive accumulation in the kidneys. The primary mechanisms underlying cisplatin-induced nephrotoxicity are related to (i) increased production of ROS from damaged mitochondrial DNA [78], (ii) activation of apoptosis in damaged renal proximal tubules [79], (iii) accumulation of cisplatin in renal tubules due to its affinity for copper transporters and organic cation transporter-2, the major influx transporters for cisplatin [80] (iv) inhibition of Na^+^/K^+^-ATPase pump, and (v) inflammation and vascular injury in the kidneys [102]. Depending on the level of exposure to cisplatin, renal tubular cells may undergo necrosis or apoptosis, with inflammatory responses further exacerbating kidney damage.

Interestingly, protein kinase Cδ (PKCδ) is a critical mediator of cisplatin-induced injury and cell death in renal cells. Inhibition of PKCδ has been found to enhance the chemotherapeutic effects of cisplatin in several tumor models while reducing its nephrotoxic side effects. PKCδ is not essential for the survival of normal cells, but it is required for the survival and proliferation of cancer cells under oncogenic stress. Therefore, inhibition of PKCδ leads to cell death in tumors but spares normal cells. Natural inhibitors of PKCδ, such as quercetin (50 mg/kg), can directly target PKCδ and Janus kinase 2 (JAK2) in the kidney, providing protective effects against cisplatin-induced nephrotoxicity (4 mg/kg) and inflammation [103,104]. This action may be mediated through the activation of Nrf2, resulting in increased levels of HO-1, which decreases TNF-α, iNOS, and IL-6 [105]. In addition, propolis-derived flavonoids and polyphenols that act as HO-1 inducers also maximize cellular intrinsic antioxidant potential. Morales et al. [106] suggest that the renal protection exerted by quercetin is due to its ability to increase metallothionein levels in the kidneys. Components of propolis may also modulate the antioxidant defense system by altering the activity of detoxification enzymes (phase I and II) and increasing the expression of γ-glutamylcysteine synthetase, which is the rate-limiting enzyme for GSH synthesis and crucial for the induction of xenobiotic responsive elements in CYP 450 genes [107]. Treatment with kaempferide has been shown to significantly ameliorate ischemia-reperfusion-induced renal injury in vitro and reduce cisplatin-induced AKI in vivo [101].

Numerous flavonoids are known to alleviate cisplatin-induced renal dysfunction by inhibiting oxidative and nitrosative stress, lowering creatinine (Cre) and blood urea nitrogen (BUN) levels, downregulating activity of MAPK and NF-κB signaling pathways, reducing inflammatory response, activating the Nrf2 and HO-1 pathways, and attenuating apoptosis [98,99,100,101,102,103,104]. Notable flavonoids that exhibit these protective effects against cisplatin-induced renal damage include naringenin, quercetin, silymarin, icariin, breviscapine, epicatechin, epicatechin gallate, daidzein, xanthohumol, sappanone A, morin and its hydrate, baicalein, and apigenin [98,99,100,101,102,103,104]. Additionally, wogonin significantly inhibits receptor-interacting protein kinase 1-mediated necrosis and the canonical WNT pathway (WNT/β-catenin pathway), providing protection against cisplatin-induced nephrotoxicity [101].

Polyphenols also modulate adaptive responses in the cochlea exposed to cisplatin and act as cardioprotectors and inhibitors of neurodegeneration. These adaptive effects may result from the interplay between cell defenses, inflammatory molecules, and key signaling pathways, including STAT-3, NF-κB, p53, and Nrf-2 [101,108].

Fetoni et al. [97] have shown the otoprotective and chemosensitizing properties of polyphenols like curcumin and ferulic acid in mitigating cisplatin-induced cochlear damage by upregulating Nrf-2/HO-1 pathway and downregulating p53 phosphorylation. Research by Lee et al. [109] indicated that low doses of resveratrol provide partial otoprotection against cisplatin-induced ototoxicity, likely through anti-oxidative (CYP1A1 and RAGE) and anti-inflammatory (NF-κB, IL-6, and IL-1β) mechanisms. However, high doses of resveratrol triggered an inflammatory response and failed to reduce cisplatin-induced ototoxicity [109].

In conclusion, polyphenols/flavonoids, including resveratrol, possess specific antioxidant, anti-inflammatory, and anti-apoptotic properties [110] that help regulate pathways associated with cisplatin-induced kidney damage. The molecular mechanisms underlying cisplatin-induced nephrotoxicity are comprehensively reviewed in the following studies [92,97,101].

Resveratrol can overcome multidrug resistance and, when combined with cisplatin, enhances chemosensitivity, reduces cytotoxicity on healthy cells, and modulates immune responses by affecting cytokine secretion, immune checkpoints, NF-κB, AKT-mTOR signaling, and NK-cell receptors [111,112]. Yang et al. [112] found that resveratrol and cisplatin synergistically reduce the viability, migration, and invasion of MDA-231 breast cancer cells by regulating EMT, PI3K/AKT, Smad, NF-κB, JNK, and ERK expression in tumor tissue. Resveratrol enhances cisplatin’s anticancer effects by promoting apoptosis, cytochrome c release, and altering Bcl-2 and Bax protein levels [113,114]. Zhang et al. [115] reported that resveratrol pretreatment reduces HIF-1α and VEGF expression in tongue and hepatoma cancer cells, affecting glucose uptake, lactate production, and Akt/mTOR signaling, while also enhancing p53 acetylation and target gene expression involved in cell death [112,115,116].

Resveratrol (50 mg/kg for 5 days) and cisplatin (2.5 mg/kg on days 10 and 12, 5 mg/kg on day 15) showed anti-angiogenic effects by inhibiting VEGF and MMP-2 activity, potentially through MMP-9-mediated angiostatin production [117]. Cisplatin stimulates macrophage activation, inducing pro-inflammatory signals like NO, TNF-α, IL-1, IFN-γ, and IL-12, leading to M1 polarization [118,119], and promotes DC maturation, which enhances T-cell activation by downregulating PD-L1/PD-L2 [118]. Resveratrol induces immunogenic cell death by increasing CRT and HMGB1 expression in ovarian cancer cells, reducing tumor volume, suppressing TGF-β, and boosting IL-12 and IFN-γ levels [116,120,121]. It also prevents PD-1/PD-L1 interactions, enhancing T-cell-mediated cancer cell death. Synergistic effects of resveratrol with other chemotherapies, including docetaxel, paclitaxel, and doxorubicin, have been confirmed in breast cancer studies [113,114].

Cancer stem cells (CSCs) may be the source of all tumor cells, contributing to chemotherapeutic resistance and metastasis. Hallmarks of CSCs include self-renewal, enhanced EMT capability, drug resistance mediated by high expression of such as ABCB1, ABCG2, and ABCB5, and tumorsphere formation with upregulated proteins Wnt, Hedgehog, and Notch. Inhibition of ABC transporters by flavonoids can prevent multidrug resistance and destroy CSCs. Flavonoids such as quercetin, luteolin, apigenin, genistein, wogonin, myricetin, fisetin, and EGCG have the potential to target CSCs in various cancers, including breast cancer, brain tumors, lung cancer, colon cancer, and melanoma (see review [64,122]). Evidence suggests that breast cancer stem cells develop resistance through upregulation of stemness and chemo-evasion markers like SOX2, OCT4, NANOG, MDR1, and CD44, following chemotherapy. The combination of kaempferol and verapamil effectively targets chemoresistance pathways in breast CSCs; this combination not only inhibits proliferation but also downregulates the expression of key genes at the RNA and protein levels, with enhanced efficacy in combined treatment. The combined treatment induces G2/M cell cycle arrest and disrupts the physical association of CD44 with NANOG and MDR1 in MDA-MB-231 cells, thus overcoming breast cancer resistance and promoting cancer cell death [123,124]. Kaempferol has also been shown to suppress breast cancer metastasis by decreasing the expression and overall activity of MMP-9, MMP-1, and MMP-3, while increasing the expression of γH2AX, a sensitive marker of DNA double-strand breaks.

##### Temozolomide

The study by Žukovska et al. [125] demonstrated that propolis (10–100 μg/mL) can alter the anticancer effects of temozolomide (10–100 μM) in U87MG human glioblastoma cell line, resulting in a nearly twofold reduction in NF-κB nuclear localization. Additionally, quercetin has been found to enhance the sensitivity of U87 and U251 human glioblastoma cells to temozolomide, an oral alkylating chemotherapeutic agent, by inhibiting heat-shock protein 27 [126]. More recently, Desai et al. [127] reported that the combination of the isoflavone biochanin A with temozolomide increases anticancer activity against glioblastoma U87 and T98G cells. This is attributed to enhanced expression of phosphorylated p53 and decreased cell viability, as well as downregulation of cell survival proteins such as EGFR, p-ERK, p-Akt, c-myc, and membrane type-1 MMP (MT1-MMP). These proteins, commonly expressed in various tissues, show broad substrate specificity, targeting numerous extracellular matrix proteins. Many flavonoids, including chrysin, diosmin, apigenin, hypericin, kaempferol, kaempferide, and naringenin, have the ability to increase the cytotoxicity of temozolomide in BCRP-overexpressing (BCRP-MDCKII) cells by reducing BCRP-mediated temozolomide efflux [106]. When combined with flavonoids, temozolomide also induces a metabolic shift from anaerobic to aerobic energy consumption and causes cell cycle arrest in the G1 phase.

#### 3.1.3. Effectiveness of Propolis in Interactions with Cytotoxic Antibiotics and Related Substances

Anthracyclines are a group of natural products isolated from soil bacteria of the *Streptomyces* genus, with over 2000 chemical variants. These anti-cancer drugs work through various mechanisms, including intercalation between DNA base pairs, which disrupts the synthesis and/or function of nucleic acids. Some anthracyclines induce DNA strand breaks and inhibit the enzyme DNA-topoisomerase II [128]. Actinomycin D, another cytotoxic antibiotic, inhibits mRNA synthesis and, subsequently, protein synthesis by stabilizing topoisomerase I-DNA covalent complexes and blocking RNA chain elongation by RNA polymerase [129]. Actinomycin D, as a DNA-interacting transcription blocker, induces apoptosis in cancer cells by inhibiting the transcription of anti-apoptotic genes [128,129].

The main group of antineoplastic substances isolated from the fungus *Streptomyces peucetius* includes the anthracyclines group, with hundreds of structural analogs synthesized to date, but only daunorubicin, doxorubicin, idarubicin, and epirubicin are currently in clinical use [128,129].

##### Doxorubicin

Anthracyclines, including doxorubicin, are among the most effective and widely used cytotoxic agents for treating solid and hematopoietic tumors. The primary anticancer mechanism of doxorubicin involves DNA intercalation, DNA strand breakage, and topoisomerase II inhibition, which subsequently promotes growth arrest and apoptosis. Doxorubicin toxicity is related to its interaction with iron, leading to ROS formation and consequent damage to cellular biomacromolecules. Excess ROS leads to oxidative stress, DNA damage, and lipid peroxidation, ultimately triggering apoptosis. Beyond typical chemotherapy side effects, doxorubicin can cause severe and life-threatening cardiac problems, potentially occurring at any point during or up to a year after treatment. Various molecular mechanisms have been proposed to explain doxorubicin-induced cardiotoxicity, including oxidative stress, topoisomerase II inhibition, mitochondrial dysfunction, dysregulation of Ca^2+^ homeostasis, intracellular iron accumulation, apoptosis and necrosis, autophagy, and myofibrillar disarray and loss [130]. Doxorubicin toxicity is exacerbated by its preferential metabolic conversion to doxorubicinol via cytoplasmic NADPH-dependent aldose and aldehyde and carbonyl reductases [130].

The synergistic effects of propolis and doxorubicin have been confirmed in various cancer cell lines, including MDA-MB-231 breast cancer cells, MCF7 breast adenocarcinoma cells, NCI-ADR/RES ovarian cancer cells, LoVo colon cancer cells, and A549 lung cancer cells [5,131,132,133,134,135]. Several molecular mechanisms for this synergistic action have been suggested: (i) disruption of the p53/ATM-regulated non-homologous end-joining pathway and double-strand breaks repairs, involving overexpressed BRCA1 and suppressed RIF1 proteins; (ii) induction of apoptosis through the regulation of pro-apoptotic (p27, PON2, catalase) and anti-apoptotic (XIAP, HSP60, and HIF-1α) proteins; (iii) propolis’s antioxidant properties promoting antioxidant-related apoptotic pathways; and (iv) decreased the expression of genes encoding MRP1 and NAD(P)H quinone oxidoreductase 1 (NQO1), sensitizing cancer cells to chemotherapy.

Cuban propolis is categorized as red, yellow, or brown based on its composition, with brown propolis being the most studied for its biological properties, which are attributed to nemorosone, a polyisoprenylated benzophenone. It has been demonstrated that Cuban propolis and its main component, nemorosone, can sensitize doxorubicin-resistant human colon carcinoma cells (LoVo Dox) compared to sensitive LoVo cells. Frión-Herrera et al. showed that brown Cuban propolis and nemorosone exhibit synergistic antiproliferative and cytotoxic effects with doxorubicin by inducing cell cycle arrest and apoptosis through increased ROS production and substantial alteration of the mitochondrial membrane potential (ΔΨm) [135]. In the animal model, the combination of propolis- and doxorubicin-induced cell cycle arrest in the S phase increased cell death and inhibited the P-gp efflux pump, increasing the intracellular concentration of doxorubicin in cancer cells. Propolis-derived components, such as quercetin, ferulic acid, CAPE, propolone B and propolonone A (from red propolis), and nemorosone (from brown propolis) may also modulate the MDR in cancer cells by inhibiting P-gp expression [135].

Certain flavonoids, including chrysin, diosmin, amentoflavone, apigenin, biochanin A, genkwanin, hypericin, kaempferol, kaempferide, licochalcone A, and naringenin, significantly inhibited BCRP in BCRP-overexpressing (BCRP-MDCKII) cells. These effects were associated with reduced BCRP-mediated doxorubicin and temozolomide efflux and increased cytotoxicity of these drugs [136]. Furthermore, luteolin has been shown to enhance the sensitivity of MDA-MB-231 breast cancer cells to doxorubicin [137] and paclitaxel [138] by inhibiting Nrf2 signaling and blocking STAT3, respectively. Luteolin causes degradation of Nrf2 mRNA and the expression of downstream antioxidant response element (ARE)-driven genes, such as NQO1, HO-1, and AKR1C [139], and induces cell death when combined with oxaliplatin, bleomycin, and doxorubicin. Similarly, in triple-negative breast cancer (TNBC) cells, luteolin-loaded nanoparticles reduced mRNA levels of Nrf2, HO-1, and MDR [139]. Furthermore, it has been shown that luteolin enhances the chemosensitivity of osteosarcoma cells and xenograft models to doxorubicin and cisplatin. It increases miR-384 levels and downregulates the pleiotrophin (PTN) expression, a heparin-binding growth factor involved in cellular differentiation, proliferation, and metastasis, which promotes chemoresistance in osteosarcoma cells by upregulating P-gp via the anti-anaplastic lymphoma kinase (ALK)/ glycogen synthase kinase 3β (GSK3β)/β-catenin signaling pathway. Luteolin reverses MDR in osteosarcoma cells and may be a promising therapeutic agent for chemoresistant osteosarcoma by inhibiting the PTN/β-catenin/MDR1 signaling axis through miR-384 upregulation [140].

Nobiletin has demonstrated cytotoxic effects against various cell lines, including those with diverse phenotypes resistant to established anticancer drugs (e.g., P-gp, ABCB5, p53, EGFR). Cross-resistance profiling with 83 standard anticancer drugs indicated a correlation with antihormonal anticancer drugs, likely due to the phytoestrogenic properties of nobiletin [141]. Adham et al. [141] found that the combination of nobiletin and doxorubicin synergistically killed HEK293-SOX5 cells, indicating that SOX5 could be a potential target in personalized treatment protocols.

Additionally, flavonoids such as quercetin, chrysin, genistein, biochanin A, kaempferol, and naringenin can act as chemosensitizers to reverse MDR by modulating Wnt/β-catenin signaling in drug-resistant KBCHR8-5 cells. Flavonoids such as quercetin, rutin, theaflavin, epicatechin-3-gallate, and tamarixetin, significantly increased doxorubicin-induced cell death in KBCK R 8-5 and MCF7/ADR cell lines by modulating levels of Wnt/β-catenin signaling proteins [49].

Quercetin has been shown to significantly inhibit the formation of doxorubicinol, the major metabolite of doxorubicin. Additionally, flavonoids such as quercetin, rutin, luteolin, apigenin, hesperidin, anthocyanin, and naringenin play an essential role in mitigating cardiac toxicity through multiple mechanisms, including reducing ROS, preventing lipid peroxidation, stabilizing mitochondria permeability and suppressing apoptosis [8,93,141,142,143,144,145,146,147]. As mentioned previously, the dose-limiting side effect of doxorubicin is its potential to cause acute and chronic cardiotoxicity. Li et al. [26] reported that isoorientin alleviates doxorubicin-induced cardiac injury by activating MAPK, Akt, and caspase-dependent signaling pathways. The cardioprotective effect of isoorientin was further supported by the downregulation of cleaved caspase 3 and upregulation of Bcl-xl. Proteomic analysis of cardiomyocytes revealed that isoorientin exerted protective effects in mouse models by targeting proteins such as caspase-3, EGFR, MAPK1, estrogen receptor 1 (ESR1), CDC42, STAT1, JAK2, lymphocyte cell-specific protein tyrosine kinase (LCK), and CDK2. Moreover, isoorientin upregulated Nrf2 and TGF-β3 while downregulating levels of phosphorylated JNK and p38 proteins, as well as Akt and Stat3 expression levels.

Propolis has also been shown to reduce cardiotoxicity. Chopra et al. [143] demonstrated that propolis (50 and 100 mg/kg) has a cardioprotective effect in doxorubicin-induced experimental cardiotoxicity. Doxorubicin (10 mg/kg) treatment elevated levels of creatine phosphokinase (CK), aspartate aminotransferase (AST), GSH, and thiobarbituric reactive substances (TBARS) in the heart, while propolis administration significantly reduced these markers by mitigating oxidative stress, inflammation, and apoptosis [144,145].

In addition to cardiotoxicity, doxorubicin causes damage to other organs. Thus, it also leads to neurotoxicity, hepatotoxicity, and nephrotoxicity. It has been shown that doxorubicin-induced ROS, such as hydroxyl radicals, superoxide radicals, and hydrogen peroxide (H_2_O_2_), contribute to nephrotoxicity, glomerular and tubular damage, and increased serum creatinine and urea levels [93,143]. Propolis has been shown to significantly reduce chromosomal damage caused by doxorubicin-induced high levels of ROS. The main protective mechanism of propolis against doxorubicin toxicity across multiple organs involves oxidative stress reduction and scavenging of reactive radicals, reduced MDA formation, and increased endogenous mechanisms of antioxidative defense (catalase, SOD, GSH). As a potent antioxidant rich in flavonoids, propolis protects cell membranes against lipid peroxidation. Compounds in propolis, such as CAPE, can inhibit ROS production and preserve the function of antioxidant systems by upregulating the activity or expression of Nrf2, which is released from its repressor (Keap1) under oxidative or xenobiotic stress [5]. Once released, Nrf2 binds to the ARE in the promoter region of cytoprotective genes and induces the expression of free radical-scavenging enzymes [5].

Furthermore, Ali et al. [146] showed that propolis alleviates elevated creatinine and urea levels in doxorubicin-induced nephrotoxicity in rats. The combined use of propolis and doxorubicin can reduce MDA levels and peroxidative damage to rat mitochondria. Propolis also effectively reduces oxidative stress caused by chemotherapeutic agents in the kidney, liver, and testis [8,133,146,147].

The protective role of WSDP in combination with doxorubicin on spleen weight and spleen and bone marrow cellularity has also been observed. Additionally, WSDP administration prevented thrombocytopenia, a common side effect in animals treated with chemotherapeutic agents [8,17,52]. Moreover, propolis significantly reduced oral mucositis, a major side effect of chemotherapy. It has been shown to be safe and effective in reducing the symptoms of oral mucositis in breast cancer and head and neck cancer patients undergoing chemotherapy and radiotherapy [148,149,150].

##### Epirubicin

Epirubicin is also a member of the anthracycline family of chemotherapy drugs [151]. It is widely used in the treatment of various types of breast cancer [151,152]. Epirubicin intercalates between DNA base pairs, disrupting the synthesis of DNA and RNA [151,152]. It also induces DNA cleavage through the action of topoisomerase II, ultimately leading to cell death. Epirubicin has been shown to induce oxidative stress by significantly decreasing hepatic antioxidant enzymes, such as glutathione peroxidase, catalase, and SOD, while increasing MDA and liver parameters (aspartate transaminase (AST), alanine transaminase (ALT), γ-glutamyl transferase, alkaline phosphatase) compared to control. Histological studies have revealed substantial liver damage, including cell necrosis, hepatic degeneration, dense lymphocytic inflammatory infiltrates, and increased levels of TNF-α and prostaglandin E2 (PGE2). In contrast, pretreatment with an ethanolic extract of propolis (250 mg/kg) restored the inflammatory markers, reduced oxidative damage, and preserved liver architecture similar to that of untreated control rats [152].

The results reported by Oršolić et al. [5,8,17,19] are consistent with these findings. They demonstrated that WSDP can prevent epirubicin-induced hematological toxicity in mice bearing metastases of mammary carcinoma. A positive outcome was also observed with combined treatment of WSDP (50 or 150 mg/kg) and epirubicin (2.5 mg/kg), which improved the number of red and white blood cells in peripheral blood. In contrast, mice treated with epirubicin alone showed a significant drop in all hematological parameters by day 13 after tumor cell inoculation. Furthermore, mice administered WSDP (50 mg/kg) orally for 20 consecutive days exhibited an increased number of exogenous colony-forming units (CFUs). Treatment with WSDP enhanced the cellularity of hematopoietic tissue and increased leukocyte counts in peripheral blood, while prolonged treatment elevated the number of myeloid and megakaryocytic CFUs.

Studies have clearly shown that propolis and related flavonoids (100 mg kg^−1^) in combined treatment not only restored the count of nucleated bone marrow cells to normal levels but also normalized hemoglobin content and red blood cell (RBC) counts. When administered alone, propolis and its flavonoids did not alter the specific activities of AST, ALT, or lactate dehydrogenase (LDH), indicating no adverse effects on the pancreas, kidney, or liver, consistent with findings from other studies [142,143,144,145,146,147,148,149,150,151].

#### 3.1.4. Effectiveness of Propolis in Interactions with Topoisomerase Inhibitors and Antimitotics

Topoisomerases I and II are essential enzymes that regulate DNA topology, playing crucial roles in DNA replication, chromosomal segregation, transcription, recombination, and repair [153]. Topoisomerase inhibitors are a class of anti-cancer drugs that inhibit the action of these enzymes. These drugs include irinotecan and topotecan, which inhibit topoisomerase I (topoisomerase I cleaves one DNA strand), and etoposide and teniposide, which inhibit topoisomerase II (topoisomerase II cuts both DNA strands simultaneously). These agents bind to the DNA-topoisomerase complex and prevent DNA resealing, leading to the accumulation of DNA breaks and subsequent cell death. Doxorubicin, discussed earlier, also inhibits topoisomerase II by preventing DNA strand recombination, thus stopping DNA replication.

Microtubule-targeting drugs activate the mitotic checkpoint by disrupting the formation of the mitotic spindle [154]. Reorganization of microtubules is a critical process during mitosis, orchestrating the movement of centrosomes and the proper segregation of sister chromatids into daughter cells. Antimitotic drugs, which target microtubules, inhibit cell growth by stopping mitosis (mitotic inhibitors). Vinca alkaloids and taxanes are two major groups of anticancer drugs that target mitosis. Given the hyperproliferative nature of cancer cells, microtubules are an ideal target for cancer therapies, including treatments for lung cancer, breast cancer, neuroblastoma, rhabdomyosarcoma, acute leukemia, and Hodgkin’s and non-Hodgkin’s lymphomas. Antimitotic drugs disrupt microtubule polymerization dynamics, acting as either microtubule-destabilizing agents or microtubule-stabilizing agents [154,155,156].

Microtubule-destabilizing agents, such as colchicine and vinca alkaloids (vincristine and vinblastine, and their semisynthetic derivatives vindesine and vinorelbine), inhibit microtubule polymerization by binding to β-tubulin near the GTP-binding site (vinca domain) at the β-α-tubulin heterodimer interface. These agents interfere with tubulin, preventing microtubule formation and leading to mitotic arrest and subsequent induction of apoptosis or a senescence-like G1 state. Microtubule-stabilizing molecules, such as paclitaxel, docetaxel, cabazitaxel, and many others, inhibit microtubule depolymerization by binding to β-tubulin. This induces mitotic arrest and activates caspase-dependent apoptosis through the Bcl-2 protein family.

##### Irinotecan

Irinotecan hydrochloride (CPT-11) is a camptothecin derivative that demonstrates antitumor activity against a range of solid tumors, including colorectal, gynecologic, colon, lung, pancreatic, breast, and ovarian cancers, as well as various forms of leukemia. After administration, irinotecan undergoes extensive liver metabolism, producing several metabolites. It is enzymatically converted by carboxylesterases into 7-ethyl-10-hydroxycamptothecin (SN-38), a metabolite with cytotoxic activity 100 to 1000 times greater than that of irinotecan itself [157,158]. SN-38 exerts its anticancer effects by binding to the topoisomerase-I-DNA complex, leading to double-strand DNA breaks and inhibition of DNA replication and transcription [157]. SN-38 is subsequently inactivated through conjugation by uridine diphosphate glucuronosyltransferases, forming the β-glucuronide derivative SN-38G [157,158]. Other metabolites of irinotecan are produced via cytochrome P450 3A4-mediated oxidation of the terminal piperidine ring [158].

Our studies [30,31,32] demonstrated that the combination of WSDP, EEP (100 mg/kg), naringin, and quercetin (50 mg/kg) with irinotecan effectively delayed the growth of the Ehrlich ascites tumor (EAT) and increased the lifespan of EAT-bearing mice. Specifically, the combined treatment of EEP and WSDP with irinotecan extended the median survival time to 59 and 70 days, respectively, compared to 39 days in the control group or the group treated with irinotecan alone. Analysis of the total cell count in the peritoneal cavity of mice revealed that all experimental groups treated with tumor cells and WSDP or polyphenolic compounds from propolis had significantly fewer cells in the peritoneal cavity than the control group. When irinotecan was combined with quercetin or naringin, the total number of cells in the peritoneal cavity was reduced by 88% and 81%, respectively, compared to the control group, and by 28% or 21%, respectively, compared to treatment with irinotecan alone [30,91]. Interestingly, pretreatment with propolis or flavonoids before irinotecan administration reduced the frequency of irinotecan-induced micronuclei (MN). However, in tumor-bearing mice, quercetin and EEP increased the number of micronucleated cells [32].

Propolis preparations and related flavonoids exhibited significant immunomodulatory effects and appeared to reduce the toxic and genotoxic effects of irinotecan on normal cells without affecting its cytotoxicity against EAT cells. The precise mechanisms by which these components interact with irinotecan are not fully understood, but several hypotheses have been proposed [32]: (i) maintaining high circulating levels of irinotecan by inhibiting P-gp efflux, (ii) enhancing selective delivery of irinotecan to tumor cells, (iii) boosting immune response and inducing direct DNA damage through apoptosis, (iv) altering the metabolic activation of irinotecan, and (v) the synergistic action of flavonoids and chemotherapeutic agents on topoisomerase I and II.

Our research reveals that pretreating tumor-bearing mice with propolis and related compounds like naringenin or quercetin, combined with irinotecan, led to several key outcomes: (i) reduced total tumor cell count in the peritoneal cavity, (ii) increased white blood cell (WBC) levels, (iii) boosted activity of macrophages and polymorphonuclear neutrophils (PMNs), (iv) protection of liver and kidney cells from irinotecan toxicity, and (v) a reduction in micronucleated cells in peripheral blood [31,32,159,160]. These observations are consistent with those reported by Wei et al. [161] and Ballester et al. [162], who suggested that flavonoids might be beneficial for mitigating undesirable effects, such as diarrhea, by inhibiting intestinal secretion and motility, reducing chronic inflammatory damage, protecting against oxidative stress, and preserving mucosal function. Proposed mechanisms for the intestinal anti-inflammatory effects of flavonoids include (i) antioxidant and antiradical properties, (ii) modulation of NO metabolism, (iii) inhibition of lipoxygenase and reduction of leukotriene B4 (LTB4) production, iv) suppression of proinflammatory cytokine production, (v) preservation of colonic absorptive function, and (vi) increasing intestinal GSH content when administered orally to normal rats [5].

Propolis and its associated flavonoids have been shown to significantly increase WBC and RBC counts in the peripheral blood of mice treated with irinotecan in a short period. When combined with irinotecan, flavonoids not only reduced drug-induced toxicities but also normalized elevated levels of liver enzymes ALT and AST, which are markers of organ dysfunction and cellular injury, typically increased by irinotecan alone [30,31,32,163]. The protective effects of propolis and flavonoids against liver and kidney dysfunction were found to be more effective in preventive treatments than in curative ones [30,31,32,163].

Díaz-Carballo et al. [164] studied plukenetione A, a novel compound derived from Cuban propolis, revealing its anti-metastatic effects in mice and notable cytotoxicity against both drug-sensitive and drug-resistant human tumor cell lines. Resistance to chemotherapeutic agents was developed in various wild-type cell lines (some examples are HT29, HCT8, MCF-7, M51, LAN-1, and LANCap) by exposing them to increasing concentrations of drugs such as adriamycin, cisplatin, etoposide, SN38, raltitrexed, and 5-FU. Plukenetione A triggered G0/G1 cell cycle arrest and DNA fragmentation in colon carcinoma cells, inhibited both topoisomerase I and DNA polymerase activities, and repressed the expression of topoisomerase II-β, EGFR, and MDR1 phenotype genes. This suggests that the anti-tumor effects of plukenetione A are primarily due to its action on topoisomerase I and DNA polymerase.

Furthermore, the prenylated flavonoid xanthohumol, derived from hops, has been found to act as a chemosensitizer for SN38 (the active metabolite of irinotecan) in resistant colon cancer SW480 cells. As mentioned, SN-38, widely used in treating metastatic colorectal cancer, can be converted into its inactive form, SN-38 glucuronide (SN-38G). However, intestinal β-glucuronidase can reconvert SN-38G back into its active form, SN-38, restoring its efficacy. In another study, the flavonoids wogonin and chrysin, when administered orally, were shown to mitigate irinotecan-induced intestinal damage, diarrhea, mucositis, and weight loss. Histological studies confirmed that flavonoids help preserve healthy villi in the ileum and colon. When combined with irinotecan, flavonoids also reduced the expression of inflammatory cytokines (IL-1β, IL-18, TNF-α, and IFN-α) and doubled the expression of tight junction proteins ZO-1 and occludin in the small intestine [165]. Quercetin may also counteract irinotecan-induced side effects. Co-administration of quercetin, which acts as a P-gp inhibitor, can enhance the oral bioavailability of irinotecan and alleviate irinotecan-induced diarrhea. Compared to existing P-gp inhibitors, quercetin is safer and presents an ideal candidate for improving the oral delivery of anticancer drugs [166].

##### Paclitaxel

Paclitaxel, known by its chemical formula C_47_H_51_NO_14_, is a tricyclic diterpenoid classified as an anti-neoplastic, antimitotic, and anti-microtubule agent used to treat various cancers, including breast, colorectal, esophageal, lung, cervical, and prostate cancers. Studies have demonstrated that combining paclitaxel with different flavonoids results in a synergistic effect in cancer treatment, both in vitro and in vivo [167]. Paclitaxel works by promoting the assembly of tubulin into microtubules, preventing their depolymerization, thereby halting cell cycle progression in the late G2 or M phase. Despite its effectiveness, common side effects of paclitaxel include hair loss, allergic reactions, nausea, vomiting, bone marrow suppression, neutropenia, leukopenia, anemia, joint and muscle pain, mucositis, weakness, and neuropathy.

Quercetin enhances the efficacy of several chemotherapeutic drugs, including paclitaxel, doxorubicin, and vincristine, by downregulating P-gp and eliminating CSCs through the nuclear translocation of the Y-box binding protein (YB-1). YB-1 is involved in numerous DNA and RNA-dependent processes, including DNA repair and mRNA regulation, and is associated with cell proliferation, differentiation, and drug resistance. Suppressing P-gp expression and reducing the number of CSCs, particularly in breast cancer cells, could reverse MDR [168]. Quercetin significantly inhibits YB-1 nuclear translocation, thereby increasing the chemosensitivity of MCF-7 and MCF-7/dox cells to various chemotherapeutic drugs.

Polyphenolic flavonoids such as chrysin, genistein, biochanin, quercetin, kaempferol, and naringenin appear to act as chemosensitizers, capable of reversing MDR by modulating Wnt/β-catenin signaling in drug-resistant KB-ChR-8-5 cells. Additionally, flavonoids like theaflavin, quercetin, rutin, epicatechin-3-gallate, and tamarixetin, significantly enhanced doxorubicin-induced cell death in KB-ChR-8-5 and MCF7/ADR cell lines through the Wnt/β-catenin signaling proteins [49].

CSCs contribute to drug resistance and cancer relapse, largely due to their capacity for self-renewal and differentiation into diverse cancer cell lineages. Therefore, suppressing P-gp expression and reducing the population of breast cancer stem cells, characterized by high levels of YB-1 nuclear protein, P-gp, and CD44^+^/CD24^−^/^low^ phenotype, which are resistant to various chemotherapeutic drugs such as paclitaxel, doxorubicin, and vincristine, could potentially reverse MDR in breast cancer cells [44,66].

Quercetin (20 µM) also enhances the therapeutic effects of paclitaxel (5 µM) in prostate cancer cells through ER stress induction and ROS production, with synergistic effects confirmed in both in vitro and in vivo models (quercetin 50 mg/kg and paclitaxel 5 mg/kg) [169], as well as in docetaxel-resistant prostate cancer (LNCaP/R, PC-3/R) cells in vitro and in a prostate cancer xenograft model in vivo [169]. Similar pharmacokinetic studies have shown that oral intake of flavones (10–20 mg/kg) significantly enhanced the bioavailability of paclitaxel in rats, potentially due to the inhibition of cytochrome P-450 (CYP 3A) and the ABCB1 efflux pump in the intestinal mucosa. Ji et al. [170] demonstrated the involvement of ERBB2 (*HER2*, receptor tyrosine kinase group of epidermal growth factor receptors), BIRC5 (survivin, baculoviral inhibitor of apoptosis repeat-containing 5), and CASP3 in the process of quercetin-paclitaxel synergy-induced apoptosis in ovarian cancer. This combination can promote cell apoptosis and inhibit cell invasion and migration through downregulation of *ERBB2* and *BIRC5* expression and upregulation of *CASP3* expression. Comparable effects were achieved with naringenin and paclitaxel in rats, while icariin alleviated paclitaxel-induced neuropathic pain via SIRT1 activation [23,167,168,169,170,171,172,173]. Propolis components, such as artepillin C, kaempferol, kaempferide, dihydrokaempferide, and isosakuranetin, were shown to contribute to the CYP450 inhibitory activity in human hepatocytes [170,172,173]. Contrary to findings in hepatic cells, a study by Cusinato et al. reported no significant effects of propolis extract on these enzymes in healthy volunteers [174].

Research by Erdogan et al. [175] indicated that naringin, a flavonoid found in grapefruit and other citrus fruits, can enhance the cytotoxic effects of paclitaxel in prostate cancer cells by inducing apoptosis via the intrinsic pathway and causing G1 phase arrest. It also upregulates PTEN expression, a major negative regulator of the PI3K/Akt signaling pathway, and downregulates NF-κB, Snail, Twist, and c-Myc mRNA, suppressing cell migration and invasion. Naringenin demonstrated synergistic effects with paclitaxel, enhancing its ability to induce apoptosis by modulating the PI3K/AKT pathway and inhibiting ERK1/2, p38, and JNK in the PC-3 prostate cancer cells. Additionally, it promoted mitochondrial membrane potential (MMP) loss and ROS generation, triggering intrinsic apoptotic pathways in these cells. In the LNCaP prostate carcinoma cell line, ROS production occurred without altering MMP [176].

The use of CAPE to reduce the toxic effects of chemotherapy and radiotherapy-induced toxicity has been extensively reviewed, demonstrating its potential to mitigate the adverse effects of various chemotherapeutic agents and radiation [177]. Curcumin has been extensively studied for its ability to sensitize tumors to chemotherapeutic agents and radiation while protecting normal tissues from treatment-induced toxicity [178]. Numerous in vitro and animal studies have shown that curcumin can sensitize a broad spectrum of tumors to various chemotherapeutic agents and gamma irradiation by downregulating key growth regulatory pathways and specific genetic targets, including NF-κB, STAT3, COX-2, Akt, antiapoptotic proteins, growth factor receptors, and MDR proteins. Additionally, curcumin has been shown to protect normal organs such as the liver, kidneys, oral mucosa, and heart from the damage caused by chemotherapy and radiotherapy [178]. Similar protective effects have been observed for propolis, honey, and their flavonoid components [8,17,91,179,180,181,182,183,184,185]. The protective effects of flavonoids appear to be mediated through their ability to induce the activation of Nrf2 and induce the expression of antioxidant enzymes, scavenge free radicals, and inhibit p300 histone acetyltransferases (HAT) activity [8,17,91,179,180,181,182,183,184,185]. Propolis-loaded nanostructured lipid carriers have demonstrated potential anti-breast cancer activity in EAC-bearing mice through multiple mechanisms, including improving antioxidant status and inducing apoptosis, suppressing angiogenesis, and inhibiting inflammatory and mTOR pathways, all linked to increased miRNA-223 expression [180].

Flavonoids like apigenin have been shown to modulate multiple signaling pathways, including PI3K/AKT/mTOR, MAPK/ERK, NF-κB, JAK/STAT, and Wnt/β-catenin, all of which contribute to their anticancer properties [186]. When combined with paclitaxel, apigenin exhibits additive effects, increasing apoptosis in cervical cancer cells. Cotreatment with apigenin and paclitaxel resulted in more than 50% cytotoxicity, compared to 29% and 24% for apigenin and paclitaxel alone, respectively. Apigenin enhances paclitaxel-induced apoptosis by reducing SOD function, depolarizing mitochondrial membrane potential, and activating caspase-2, making HeLa cells more vulnerable to paclitaxel-triggered cell death [187].

The chemotherapeutic efficacy of paclitaxel against hypoxic tumors is often limited, but co-administration with apigenin can overcome hypoxia-induced resistance in cancer cells. Apigenin suppresses HIF-1α expression in hypoxic tumors by simultaneously inhibiting both the AKT/p-AKT pathway and Hsp90, thereby boosting the anticancer activity of paclitaxel [188]. The synergistic interactions between flavonoids and paclitaxel in cancer treatment are further explored in a comprehensive review [167]. Furthermore, targeting CD73, ecto-5′-nucleotidase, with flavonoids in combination with paclitaxel inhibits cancer stem cells and increases lymphocyte infiltration in a triple-negative mouse model of breast cancer (TNBC) [189]. CD73 is a novel immune checkpoint associated with adenosine metabolism that promotes tumor progression and angiogenesis by suppressing the antitumor immune response through silencing tumor-infiltrating T- and NK-cell activation while amplifying regulatory T-cell activation. This is achieved mainly by the increase of intracellular cAMP levels in tumor-infiltrating lymphocytes, inducing a release in *IL-10* and *TGF-β* immune-suppressing cytokines. Since adenosine induces immunosuppression, angiogenesis, mucosal hydration, and ischemic preconditioning, targeting CD73 and CSCs allows for increasing the efficacy of chemotherapy, overcoming treatment resistance, and improving clinical outcomes [189].

##### Docetaxel

Docetaxel is a semisynthetic taxane that has demonstrated significant prostate-specific antigen-lowering effects in men with androgen-independent prostate cancer (AIPC) [190,191,192]. Its antineoplastic activity is primarily due to two mechanisms: (i) the inhibition of microtubular depolymerization, leading to cell cycle arrest in the G(2)M phase; and (ii) the downregulation of antiapoptotic genes such as Bcl-2 and Bcl-xL, promoting apoptosis [193]. The pathways by which docetaxel induces apoptosis differ between androgen-dependent and androgen-independent prostate cancer cells, which is crucial for designing targeted therapies for both localized and advanced stages of the disease. Compared to paclitaxel, docetaxel has a higher affinity for tubulin and induces stronger apoptosis through bcl-2 phosphorylation.

P-gp is minimally expressed in normal prostate tissue, but its expression increases by 35% in tumor epithelial cells in primary prostate cancer cultures, contributing to chemotherapy resistance. P-gp expression correlates with tumor stage and grade, as well as with MRP1 [194]. According to Pastina et al. [195], genetic variations in P-gp (ABCB1) can affect the severity of docetaxel-induced neuropathy and neutropenia, overall survival, clinical outcomes, and treatment-related toxicity. Additionally, BCRP (also known as ABCG2), a stem cell marker, is associated with prostate cancer. The C421A polymorphism in BCRP is directly linked to stem cell chemoresistance and poorer patient survival rates [196,197]. Furthermore, BCRP phosphorylation by serine/threonine kinase Pim-1 has been shown to contribute to docetaxel resistance in prostate cancer cell lines [197].

Docetaxel was the first cytotoxic therapy shown to provide a survival benefit in patients with castration-resistant prostate cancer. Unfortunately, a significant number of men with this condition do not respond to docetaxel-based therapy, and all patients eventually develop resistance. Resistance mechanisms include reducing intracellular drug concentrations, counteracting the drug’s microtubule-stabilizing effects, and bypassing the cytotoxic effects of taxanes through alternative growth pathways or evading apoptosis. Studies have demonstrated that quercetin can overcome docetaxel resistance in prostate cancer cells (LNCaP/R, PC-3/R), both in vitro and in a prostate cancer xenograft model in vivo. This was achieved by reversing P-gp upregulation, reducing mesenchymal and stem-like cell phenotypes, and inhibiting androgen receptor and PI3K/Akt signaling pathways. According to Liskova et al. [49], flavonoids with high hydrophobicity and more planar structure, such as kaempferol, exhibit greater inhibitory effects on ABC transporters. Moreover, flavonoids, including quercetin, can act as mutagens, inhibitors of key regulatory enzymes, or pro-oxidant molecules in prostate cancer cell lines. For example, in DU-145 cells, quercetin increased ROS levels and decreased cell survival by inhibiting anti-apoptotic pathways, while in LNCaP and PC3 cells, which have an oxidative cellular environment, quercetin acted as a ROS quencher [198].

Flavonoid components of propolis, such as delphinidin, quercetin, and myricetin, interact with multiple signaling pathways, including the Ras/Raf/MEK/MAPK pathways, by inhibiting their activity in an ATP-noncompetitive manner. Additionally, luteolin and myricetin exert antitumor effects by directly binding to Fyn or Src proteins, non-receptor tyrosine kinases that activate the Ras/Raf/MEK/MAPK pathways at the ATP-binding site. Flavonoid components of propolis also inhibit the phosphorylation and activity of the PI3K/Akt signaling pathway by binding to ATP-binding sites and suppressing COX-2 expression, thereby reducing cell migration and the activity of metastasis-promoting proteins such as MMP-9, MMP- 13, and VEGF [199,200,201]. Targeting the PI3K/Akt/mTOR axis with flavonoids can reverse EMT and reduce the invasiveness of prostate tumor cells. It has been shown that the anti-cancer properties of propolis and related flavonoids can prevent VEGF-mediated angiogenesis in prostate cancer by downregulating VEGFR2 and inhibiting the PI3K/Akt/mTOR pathway, as well as downregulating NADPH oxidase 1 (NOX1) and HIF-1 [199,200,201]. Zughaibi et al. [202] demonstrated that diverse flavonoids (such as quercetin, myricetin, kaempferol, isorhamnetin, genistein, halangin, and hesperidin) inhibit the PI3K/Akt/mTOR pathway in various cancers, which results in inducing apoptosis, autophagy, angiogenesis inhibition, cell cycle arrest at G2/M, reduced cell migration, and reversal of drug resistance.

#### 3.1.5. Flavonoids in Combination with Receptor Tyrosine Kinase Inhibitors (RTKIs) and Serine/Threonine-Protein Kinase

Tyrosine kinases (TK) are an important group of protein kinases that play a vital role in cell signaling pathways, regulating essential cellular functions such as proliferation, differentiation, metabolism, migration, survival, and apoptosis [203]. Overexpression or mutations in tyrosine kinases can lead to uncontrolled cell proliferation and are linked to tumor invasion, metastasis, and angiogenesis. Structurally, tyrosine kinases are classified into receptor tyrosine kinases (RTKs) and non-receptor tyrosine kinases (NRTKs). To date, more than 58 RTKs, organized into at least 20 subfamilies [204], have been identified, including the EGFR, insulin growth factor (IGF)-1 receptor, platelet-derived growth factor (PDGF)-receptor and c-Kit, VEGF receptor, fibroblast growth factor (FGF)-receptor, hepatocyte growth factor receptor (HGFR/c-Met), AXL and others. NRTKs are further divided into nine subgroups: Src, Csk, Abl, Tec, Fak, Syk, Jak, Ack, and Fes kinase families.

In response to various external and internal stimuli, TKs play a pivotal role in many diseases, including cancer. As a result, therapies targeting TKs have emerged as effective strategies for treating a range of cancers. RTK inhibitors work by competing with ATP for binding to the ATP-binding site, thus inhibiting tyrosine kinase phosphorylation and subsequently blocking cancer cell proliferation.

Flavonoids have the ability to bind to the ATP-binding site of several kinases, functioning as RTK inhibitors (RTKIs). RTK inhibition through this mechanism could be a key aspect of flavonoids’ anticancer effects [205,206,207,208,209,210,211,212,213]. Studies have shown that combining RTKIs with chemotherapy, immunotherapy, and radiotherapy can enhance therapeutic efficacy and delay the development of resistance [205,206,207]. The beneficial effects of combining geftinib, sorafenib, icotinib, sunitinib, or erlotinib with various flavonoids, such as quercetin, apigenin, EGCG, kaempferol, naringenin, baicalein, wogonin, fisetin, and others, have been substantiated [208,209]. Despite these benefits, the combination of EGFR-TKIs with chemotherapy and immunotherapy has also been associated with increased toxicity [49,210,211]. While most studies indicate that flavonoid and RTKI combinations are synergistic and more effective, some flavonoids may compete with or antagonize the action of RTKIs. Flavonoids mainly inhibit signaling pathways such as MAPK, PI3K/Akt, and JAK/STAT, and when combined with RTKIs, they could significantly impact cancer treatment strategies. This section focuses on the combination of flavonoids with EGFR-TKIs and a few multi-targeted TKIs [212,213].

Studies have demonstrated that flavonoids like curcumin, chrysin, apigenin, hesperidin, kaempferol, fisetin, naringenin, and nobiletin can be effectively used in cancer combination therapy with RTKIs. Several flavonoids have been shown to inhibit the growth of carcinoma cells and/or induce apoptosis primarily by downregulating key signaling pathways, including EGFR, PDGFR, VEGFR, FGFR, and c-Kit. Tyrosine kinase inhibitors (TKIs) are generally classified into three main categories: (i) inhibitors of tyrosine kinase activity (ATP-competitive inhibitors), (ii) inhibitors of protein–protein interactions (targeting SH2, SH3, or substrate-binding domains), and (iii) enzyme destabilizers that disrupt the association between Src and its molecular partners [211]. For example, quercetin binds to the ATP-binding pocket or active site of RTKs like EGFR, VEGFR2, FGFR1, IGF1R, and c-MET through hydrogen bonds, hydrophobic interactions, and π-π interactions. In contrast, luteolin has the highest binding affinity to EGFR, inhibits EGFR autophosphorylation, and promotes EGFR degradation via the lysosomal pathway in epidermoid carcinoma (A431) and NSCLC (NCI-H1975) cells. Luteolin-induced EGFR degradation is attributed to its inhibition of Hsp90 binding to the mutant EGF receptor, thus disrupting PI3K/Akt/mTOR pathway signaling, which ultimately leads to apoptosis of NSCLC cells. The interaction between luteolin and the EGFR active site involves multiple hydrogen bonds, hydrophobic interactions, and electrostatic interactions [214]. Luteolin has been shown to inhibit the activity of several RTKs, such as IGFR, EGFR, and ERs, along with their downstream effector molecules, leading to the suppression of cancer cell growth and progression. Luteolin’s inhibitory effect extends to a broad range of RTK-related components, including VEGF, PI3K, Akt, MAPK, MMP-2, MMP-9, Fak, Bcl-2, Bcl-xL, NF-κB, COX-2, STAT-3, TNF-α, and HIF-1α [215,216,217]. Similarly, apigenin has been found to inhibit the EGFR phosphorylation and its downstream signaling pathways, such as MEK1/2 and ERK1/2, in lung mucoepidermoid carcinoma (NCI-H292) cells [218]. Apigenin and other flavonoids are capable of modulating EGFR, an attractive therapeutic target, by inhibiting Axl mRNA and protein expression, as well as Axl promoter activity, in NSCLC cells (A549, NCI-H460) [211,219]. Additionally, apigenin has been shown to downregulate the EMT-promoting transcription factors Snail and Slug in both EGFR wild-type (A549) and mutated NSCLC cells (HCC827) [220].

Resveratrol has been shown to inhibit VEGFR-induced angiogenesis by blocking Src tyrosine kinase, which phosphorylates vascular endothelial cadherin. It selectively inhibits the activation of downstream PDGFR and EGFR signaling pathways in human HaCaT, A431 cells, and MCF-7 cells. The proposed mechanism of action for resveratrol and its derivatives involves EGFR suppression, which is dependent on ERK activation in a dose-dependent manner. Additionally, compounds such as caffeic acid, CAPE, gallic acid, ferulic acid, and tannic acid have shown potential in targeting RTKs for cancer treatment, exhibiting significant effects in inhibiting the EGFR, VEGFR, and PDGFR signaling pathways (see review paper [5]). Various flavonoids, including resveratrol, gallic acid, diosgenin, timosaponin III, 3,3′-diindolylmethane, EGCC, pomegranate, curcumin, and genistein, have been found to either directly or indirectly inhibit the mTOR pathway [221].

The treatment of breast cancer varies based on molecular classification and subtype, defined by the presence of ERs, PRs, and human epidermal growth factor receptor 2 (HER2) status: hormone receptor-positive with luminal A (ER+PR+HER2−) and luminal B (ER+PR+HER2+) phenotypes, HER2-positive (ER−PR−HER2+), and triple-negative/basal-like (ER−PR−HER2−). Approximately 70% of breast cancers are ER-positive [222]. Standard breast cancer treatment involves non-specific chemotherapy, supplemented with targeted drugs based on the molecular subtype: (i) estrogen antagonists like tamoxifen, fulvestran, or aromatase inhibitors for ER-positive tumors; and (ii) anti-HER2 antibodies like trastuzumab and TKIs such as lapatinib for HER2-positive tumors. For triple-negative tumors, systemic chemotherapy with doxorubicin and paclitaxel is used, along with antibodies targeting VEGFR, such as bevacizumab, which inhibits angiogenesis. Clinical studies have shown positive results when combining docetaxel with trastuzumab and/or estramustine in patients with metastatic androgen-independent prostate cancer [24,193,223] and in patients with metastatic extramammary Paget’s disease [223]. The review by Balogun et al. [224] showed the molecular mechanisms and structures of natural compounds (flavonoids, phenolic acids, alkaloids) as RTKIs targeting vascular endothelial growth factor receptor (VEGFR), epidermal growth factor receptor (EGFR), insulin receptor (IGFR/IR) and platelet-derived growth factor (PDGFR). Furthermore, directions are given for reducing the oncogenic potential of cancer. It was also pointed out that the use of natural products as ATP-competitive inhibitors of RTKs can reduce side effects and lower toxicity associated with small molecules such as imatinib, gefitinib, and erlotinib.

The anticancer properties of various flavonoids, including genistein, daidzein, and quercetin, may be attributed not only to their antiproliferative and anti-inflammatory effects and ability to scavenge free radicals but also to their potential to mimic estrogenic activity [225]. Thus, treating BT474 breast cancer cells with 25 µM genistein for 3 days resulted in reduced expression of EGFR, HER2, and HER3. Similarly, resveratrol affected the mRNA expression of genes involved in ER signaling pathways. It exhibited greater activity as an ER agonist at low doses (10^−11^–10^−8^ M) in ER-positive breast cancer cells compared to ER-negative cells, although it may also activate other pathways that are independent of ER signaling. Furthermore, quercetin-3-O-glucuronide (Q3G), a metabolite of quercetin, suppressed cAMP production and Ras activation, which was accompanied by a decrease in ROS levels. In the MDA-MB-231 breast cancer cell line (ER−/PR−/HER2−, EGFR+), curcumin reduced ERK phosphorylation, while apigenin inhibited tyrosine phosphorylation of HER2 and reduced the expression of phospho-JAK1 and phospho-STAT3.

In summary, various polyphenols, including EGCG, genistein, quercetin, curcumin, and resveratrol, effectively decreased phosphorylation or the expression of EGFR family members and key signaling molecules such as MAPK, PKA, p38, Akt, Elk, JAK1, and STAT3 at micromolar concentrations. However, combining cisplatin with genistein or tamoxifen with genistein decreased ROS production, apoptosis, and autophagy in MCF-7 cells but not in T47D and MCF-7 overexpressing ERβ. This suggests that the administration or consumption of genistein may be harmful in breast cancers with a high ERα/ERβ ratio [226].

Furthermore, it is important to point out that cancer stem cells express elevated levels of ABCG2 (subfamilies ABC transporters involved in multi-drug chemoresistance), and inhibition of ABCG2 can prevent multidrug resistance and selectively destroy CSCs [196,227]. Flavonoids as ABCG2 inhibitors can sensitize cancer stem cells to the activity of standard chemotherapy including estrogenic compounds. Thus, has been reported that several tamoxifen derivatives, in addition to phytoestrogens and flavonoids, counteract multidrug resistance, leading to the selective killing of CSCs [66,228]. Similarly, inhibitors (TKIs), including imatinib and gefitinib, also downregulate or inactivate ABCG2 [66,229].

**Figure 3:**
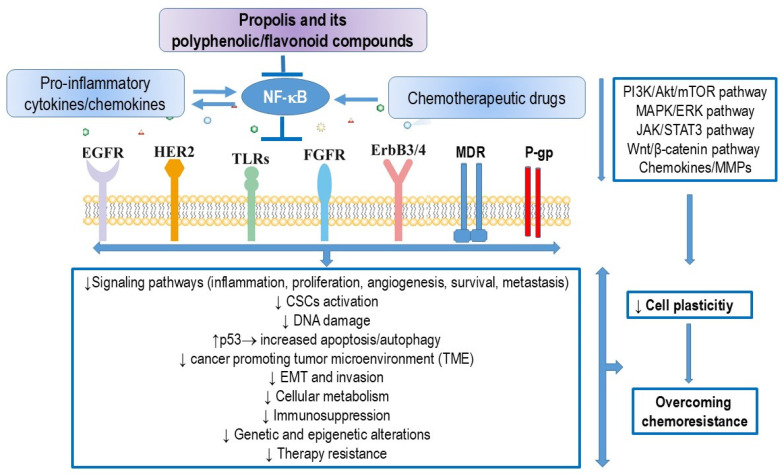
The effect of propolis and its polyphenolic and flavonoid components in the inhibition of NF-κB activation and their role in regulating proliferation, inflammation, angiogenesis, invasion, metastasis, and chemotherapy resistance. NF-κB can be activated by various factors, including reactive oxygen species (ROS), chemotherapeutic agents, ionizing radiation, TNFα, IL-1β, LPS, and viruses. Once activated, NF-κB translocates to the nucleus and regulates the transcription of genes encoding Bcl-2, Bcl-xL, XIAP, survivin, and Akt. This activation promotes cancer aggressiveness, tumorigenesis, drug resistance, and epithelial–mesenchymal transition (EMT). Propolis and its polyphenolic and flavonoid compounds can suppress acquired chemoresistance by disrupting the NF-κB-IL6 or NF-κB-ABC transporter pathways. Polyphenolic components of propolis can also help overcome multidrug resistance (MDR) to chemotherapeutic agents in various types of cancer. They achieve this through the downregulation of efflux pumps and anti-apoptotic proteins (survivin, XIAP), inhibition of the NF-κB signaling cascade, reduction in cancer stem cells progenitor formation, increased cellular uptake of chemotherapeutic agents, epigenetic mechanisms, upregulation of pro-apoptotic factors (DIABLO, APAF1), or modulation of several signaling pathways and enzymes. Abbreviations: NF-κB, Nuclear factor kappa-light-chain-enhancer of activated B cells; VEGF, vascular endothelial growth factor receptor; HER2, human epidermal growth factor receptor; TLRs, toll-like receptors; EGFR, epidermal growth factor receptor; ErbB3/4, receptor tyrosine-protein kinase 3/4; MDR, multi-drug resistance, P-gp, P-glycoprotein; EMT, epithelial–mesenchymal transition; CSCs, cancer stem cells; MMPs, matrix metalloproteinases; TME, tumor microenvironment; ↑ upregulated; ↓ downregulated.

Additionally, propolis and its polyphenolic and flavonoid compounds, which act as ATP-competitive inhibitors of RTKs, can potentially reduce the side effects and toxicity associated with small-molecule inhibitors like imatinib, gefitinib, and erlotinib. By inhibiting NF-κB, propolis and flavonoids regulate numerous signaling pathways involved in cell proliferation, inflammation, angiogenesis, invasion, metastasis, and resistance to chemotherapy (see Figure 3).

Furthermore, cancer prognosis is closely linked with the TAMs, where the M2 phenotype participates in tumor angiogenesis, tumor invasion, metastasis, immunosuppression, and cell activation. It has been shown that the blockade of the M2 phenotype, associated with pro-tumoral features, can be achieved by targeting two main transcription factors: STAT3 (sorafenib, sunitinib, WP1066, and resveratrol) and STAT6 (4-HPR, leflunomid, TMX264, and AS1217499). All these inhibitors provide tumor regression and inhibit angiogenesis [230].

## 4. Radiotherapy: Propolis and Its Polyphenolic/Flavonoid Compounds Can Protect Normal Tissue from Radiotherapy While Sensitizing Tumor Cells to Radiotherapy

### 4.1. Cancer Radiotherapy

Radiotherapy is a form of cancer treatment that uses high-energy radiation to destroy cancer cells and reduce tumor size. The biological effects of ionizing radiation are the result of a series of physical, chemical, biochemical, and cellular responses that occur after the radiation energy is absorbed by the surrounding medium. The energy from radiation is much greater than the energy in the bonds of many molecules, often leading to bond breakage and the release of secondary electrons [231]. Ionizing radiation can cause various types of damage in the body, including cell death, chromosomal aberrations, DNA damage, mutations, and cancer development [232].

Cellular damage from radiation can result from either direct or indirect effects on cell molecules. Regarding direct effects, radiation energy acts directly on DNA or other organic molecules, disrupting their structure by breaking covalent bonds, leading to the disintegration of these molecules into smaller fragments. This structural damage can result in cell injury or even cell death. Surviving damaged cells may later contribute to cancer development or other abnormalities. High linear energy transfer radiation, such as alpha particles and neutrons, and high radiation doses are often responsible for this type of damage. In contrast, indirect effects are caused by the ionization and excitation of water molecules (known as radiolysis), which leads to the production of hydrogen peroxide, molecular hydrogen (H_2_), and various highly reactive radicals, including hydrogen radicals (H•), hydroxyl radicals (OH•), hydroperoxyl radicals (HOO•), superoxide anion (O_2_^−^•), and hydrated electrons and protons. The number of free radicals produced depends on the radiation dose. These reactive species can damage cellular components, with indirect effects accounting for approximately 70% of radiation-induced cell damage [233]. Free radicals, characterized by unpaired electrons, act as strong electrophiles, i.e., strong oxidizing agents, making them highly reactive with DNA and other cellular molecules. They rapidly interact with nucleic acids, lipids, proteins, and carbohydrates, causing further damage [232].

**Figure 4:**
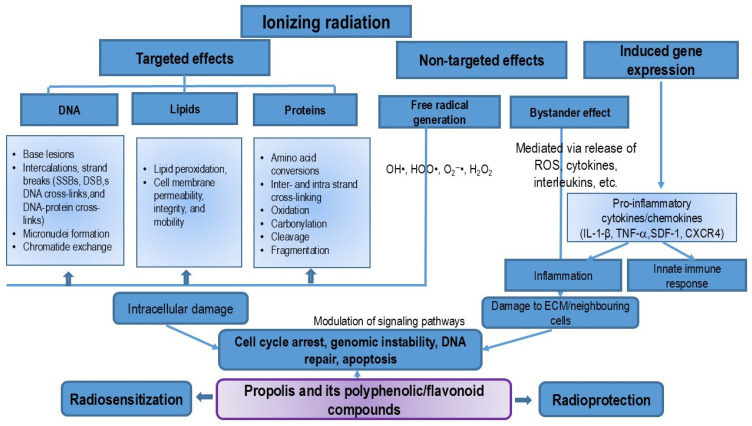
Overview of the direct and indirect mechanisms of cell death and damage due to ionizing radiation. In addition to the direct ionization of critical cellular targets, radiation induces damage indirectly by generating ROS. The indirect mechanism is responsible for over two-thirds of radiation-induced biological effects. The difference in radiation sensitivity between aerobic (normoxic) and hypoxic cells is due to the presence of oxygen. Oxygen, when present during radiation or shortly after radiation exposure, reacts with DNA radicals, causing permanent damage. Beyond oxygen-induced damage, inflammation plays a role in indirect toxicity through the production of various pro-inflammatory cytokines and chemokines (IL-1-β, TNF-α, SDF-1, TGF-β, CXCR4). These inflammatory mediators trigger long-term inflammatory responses and fibrosis (particularly TGF-β), leading to chronic inflammation and tissue injury. Propolis and its polyphenolic compound, acting as antioxidants, anti-inflammatory agents, or pro-oxidants (depending on normal or tumor cells and the tumor microenvironment), may contribute to radioprotection or enhance the effects of radiation on tissues. Abbreviations: ROS, reactive oxygen species, IL-1β, interleukin-1 beta, TNF-α, tumor necrosis factor alpha; ECM, extracellular matrix, SDF-1, stromal cell-derived factor-1, TGF-β, transforming growth factor-beta; CXCR4, CXC chemokine receptor 4.

Radiotherapy also disrupts cellular functions, affecting cell membranes, organelles, and the regulation of cellular signaling pathways. Additionally, some damage may result from the “bystander effect”, in which unirradiated neighboring cells receive signals from irradiated cells, leading to a damage response, with both immediate and long-term pathological consequences (Figure 4).

ROS play an important role in mediating cell death through multiple ways, including apoptosis, autophagy, ferroptosis, and immunogenic cell death [234]. Radiotherapy-induced ROS induce tumor cell apoptosis through the P53 pathway, mitochondrial pathway, and death receptor ligand pathway. Mitochondrial-dependent pathway acts in response to death stimuli, including DNA damage, chemotherapeutic agents, serum starvation, and radiation, by disrupting the mitochondrial permeability transition pore (mPTP) and triggering the release of cytochrome c into the cytosol [234,235]. Once released, cytochrome c binds to Apaf-1, forming an apoptotic complex that activates caspase 9, initiating a cascade of caspase activation [235]. ROS are also involved in apoptosis induced by p53 and Fas signaling pathways [235]. Additionally, DNA damage caused by ROS and RNS triggers a cascade of signaling events that activate the transcription of specific genes, potentially leading to apoptosis [235,236]. ROS also induces autophagy of tumor cells by activating AMPK, MAPK, and LC3 [234]. Ferroptosis is caused by lipid peroxidation, which is caused by iron metabolism disorder and ROS accumulation. In addition, ROS regulate the function of different cells in TME through multiple pathways, leading to immunogenic death or immune escape of tumor cells. Immunogenic cell death (ICD) is induced by the activation of an adaptive immune response in immunocompetent hosts. Oxidative stress can induce immunogenic death of tumor cells through multiple mechanisms including (i) endoplasmic reticulum (ER) stress caused by overproduction of ROS, (ii) mitochondrial pathways of ROS production, and (iii) lysosome membrane permeability (LMP). ROS can promote the release of tumor-associated antigens (TAAs), regulate the infiltration and differentiation of immune cells, antitumor immunity, and immunogenic death [234,237]. As signaling molecules, ROS regulate the expression of genes involved in immune response, proliferation control, and cellular differentiation. Therefore, an imbalance in ROS levels can disrupt diverse signaling pathways, resulting in altered gene expression [235,236,237,238].

Radiotherapy can also modulate the immune response through increased tumor antigen presentation, priming of tumor-specific cytotoxic T-cells, as well as improved T-cell homing, engraftment, and function in tumors. Mechanisms of immunomodulation induced by radiotherapy include increased recognition of “neoantigens” released from damaged tumor cells by immune cells. In addition to releasing “neoantigens” generated by radiation-induced DNA and protein damage through ROS production, tumor cells also release damage-associated molecular patterns (DAMPs) after IR, including high mobility group 1 (HMGB1), heat-shock proteins (HSP) and calreticulin (CRT), which mediate phagocytosis of antigen-presenting cells (APC) and initiate tumor-specific T-cell activation. Extracellular release of DAMPs CRT, HSPs, HMGB1, adenosine 5-triphosphate (ATP), spliceosome-associated protein 130, defensins, and S100 proteins are the main features and mechanisms of ICD [234,237].

IR can increase MHC-I expression in tumor cells, which facilitates recognition by cytotoxic T-cells. Secondly, IR activates the innate immune response and immune checkpoint upregulation through the production of type I interferon (IFN) through the stimulation of the STING-TBK1-IRF3 signal axis. Type I IFN is crucial in the activation and regulation of dendritic cell (DC) function and helper T-cell differentiation. However, STING can also induce radiation resistance and immunosuppression through upregulation of programmed cell death ligand 1 (PD-L1) to promote immune escape and through mobilization of myeloid-derived suppressor cells (MDSC) and activation of indoleamine 2,3-dioxygenase (IDO). Third, radiation regulates the immune microenvironment through increased infiltration of CD8+T cells, and IFN-γ can promote normalization of tumor vasculature and induce polarization of M2-like macrophages toward the M1 phenotype. However, IR can also induce the secretion of TGF-β, inhibit CD8+T cells, and increase the proportion of regulatory T-cells (Tregs), leading to immunosuppression in tumors. In order to enhance the antitumor effect, the combination of RT and immunotherapy has become a new strategy for cancer treatment [234].

In addition to the production of free radicals, indirect toxicity following radiation exposure is related to inflammatory processes. In vivo studies show that ionizing radiation rapidly activates macrophages and triggers an inflammatory response in the hematopoietic system [238]. Activated macrophages produce clastogenic factors, mediated by superoxide and NO, which can induce gene mutations, base modifications, DNA breaks, and cytogenetic damage in neighboring cells [238]. Shortly after irradiation, surrounding tissues initiate pro-inflammatory reactions, resulting in the release of pro-inflammatory cytokines and chemokines such as IL-1, IL-6, TNF-α, and TGF-β. These mediators drive long-term inflammatory response, leading to chronic inflammation and subsequent tissue damage (Figure 3). While free radical production is primarily responsible for short-term radiation toxicity, pro-inflammatory response is the main driver of long-term radiation-related toxicity [239]. The effects of direct and indirect actions include both biological and psychological changes, which may manifest immediately or even decades later. Genetic and epigenetic changes likely contribute to these alterations [238,239].

The response of tumor cells to radiation is influenced by five key factors, known as the “5Rs” of radiobiology: (i) repair of sublethal cellular damage, (ii) repopulation of cells after radiation, (iii) redistribution of the cells within the cell cycle, (iv) reoxygenation of the surviving cells, and (v) radiosensitivity [231,232,233].

### 4.2. Radioprotective Effect of Polyphenolic/Flavonoid Compounds on Normal Tissue

An ideal radioprotective agent should repair DNA and cell damage, regenerate tissues, modulate immune response, scavenge free radicals, reduce oxidative stress, have minimal drug interactions, protect against radiation with minimal side effects, be stable, be easy to administer, be affordable, reach target organs effectively, and cross the blood–brain barrier when needed [239,240,241,242].

Propolis and its flavonoids exhibit radioprotective properties by scavenging free radicals, upregulating Nrf2, boosting antioxidant enzymes, increasing GSH levels, and inhibiting pro-oxidant enzymes like NADPH oxidase [5,6,7,8,242,243,244,245,246,247,248,249,250,251,252]. They also promote DNA repair, prevent apoptosis, and reduce radiation-induced damage by inhibiting pathways like TNF-α, NF-κB, and AP-1 [242,243,244,245,246,247,248,249,250,251,252].

Propolis and its flavonoids enhance DNA repair via base excision, nucleotide excision, mismatch repair, and double-strand break repair (homologous recombination and non-homologous end joining), reducing genetic instability [235,239]. Furthermore, they stabilize DNA structure, interact with phosphate groups, and promote cell survival by increasing Bcl-2 and reducing Bax/Bak levels. They also inhibit inflammation and fibrosis by blocking NF-κB and cytokines like TNF-α, IL-1, IL-6, and TGF-β [240,249,252].

It has been reported that short-term treatment with quercetin has antioxidative and antiapoptotic effects, whereas prolonged treatment leads to pro-oxidative and pro-apoptotic outcomes [240]. However, a human study demonstrated that topical and oral administration of quercetin helped reduce skin damage during radiotherapy in patients with head and neck cancers [231]. Several phytochemicals, including caffeic acid, quercetin, naringin, and chrysin, as well as propolis, exhibit multiple physiological effects and antioxidant activity, contributing to radioprotection in both in vivo and in vitro settings [241,242,243,244,245,246,247,248,249,250,251,252,253,254,255,256,257,258,259]. Quercetin also inhibits NF-κB activation by suppressing ERK and p38 kinase inhibition, enhancing its radioprotective properties [260]. Apigenin, genistein, and luteolin reduce NO production by inhibiting iNOS [5,261]. Flavonoids, in general, suppress the expression of enzymes and proteins associated with inflammation, partly by blocking NF-κB, AP-1, and MAPK activation [261].

Studies show that myricetin and quercetin protect against UV-induced skin damage and increase WBC counts after X-ray exposure and radiotherapy [258,259,262,263]. Propolis and flavonoids, given before or after whole-body γ-irradiation (9 Gy) in mice, reduced DNA damage, as shown by comet assays, with effects better than aminoethyl isothiourea (AET). WSDP and EEP (100 mg/kg) reduced lymphocyte DNA damage, with WSDP (54.46%) being the most effective. Post-irradiation treatment with propolis compounds also decreased DNA damage, with WSDP reducing it by 89.24% [246,247,248,251,252].

Curcumin pretreatment significantly reduced micronucleated cells, chromosomal aberrations, and SOD activity while increasing catalase in liver tissue of irradiated rats [264,265,266]. It lowered tumor incidence and improved radiation-induced oral mucositis in animal models [264,265,266,267,268,269,270,271,272,273]. Curcumin also showed radiosensitizing effects across various tumors and protected healthy tissues from radiation side effects like dermatitis and pneumonitis [265,266,267,268,269,270,271,272,273]. In wound healing, curcumin enhanced contraction, collagen synthesis, and DNA production in mice exposed to γ-radiation [270,271,272]. It reduced skin toxicity by lowering inflammatory cytokines post-irradiation [273].

Naringenin protected against radiation-induced intestinal injury in mice and human cells [274]. Propolis and flavonoids also reduced radiation-induced inflammation and promoted healing in mucositis models [5,256,258,275,276,277]. Wogonin administered orally to irradiated rats increased antioxidant enzymes and reduced inflammatory markers [278].

Propolis and quercetin also reduced γ-ray-induced DNA damage in human lymphocytes, outperforming AET with longer exposure [247]. Turkish and Croatian EEP similarly protected against radiation damage in human cells [243,258,279].

Another study with Croatian propolis demonstrated the radioprotective effects of WSDP (100 µg/mL) and its flavonoid components, including caffeic acid, chrysin, and naringin (50 µM), in human lymphocytes exposed to 4 Gy γ-irradiation [280]. The protection observed was comparable to the effects of AET, a known radioprotectant. Using chromosomal aberration, micronucleus, and comet assays, the study found no toxic effects of WDSP and its flavonoids. However, a 30-min pretreatment significantly reduced radiation-induced DNA damage. Similarly, Prasad et al. [281] reported that 30-min pretreatment with ferulic acid (1, 5, 10 µg/mL), a cinnamic acid derivative from propolis, protected human lymphocytes from γ-radiation-induced (4 Gy) MN formation, chromosomal aberrations, and lipid peroxidation. Rithidech et al. [282] found that apigenin reduced MN formation in human lymphocytes exposed to 2 Gy γ-radiation, while Zhang et al. [283] reported that morin, another flavonoid, decreased intracellular ROS and protected cells from DNA damage, lipid peroxidation, and apoptosis. Kumar and Tiku [284] showed that naringenin induced apoptosis in cancer cells but protected normal cells from radiation-induced apoptosis by modulating p53, Bax, and Bcl-2 expression. Their findings align with previous studies [242,248,251,252,285], which showed that propolis and its components, such as naringenin, inhibit the NF-κB pathway and downregulate radiation-induced apoptotic proteins, offering radioprotection at cellular, tissue, and organismal levels.

Hematopoietic syndrome, caused by radiation doses between 2.5–8 Gy, leads to the depletion of hematopoietic stem cells and mature hemopoietic and immune cells [242]. WBCs severely damaged by irradiation undergo apoptosis or necrosis. Our research demonstrated significant differences in the survival times of whole-body irradiated (WBI) mice pre-treated with test compounds compared to the control group. Quercetin was the most effective compound, offering protection comparable to AET, likely due to its chemical structure for scavenging free radicals [5]. However, post-irradiation treatment with propolis preparations (EEP and WSDP) was ineffective [246,247,248].

Propolis and its polyphenolic/flavonoid components may enhance WBI mice survival by increasing the activities of antioxidant enzymes (SOD, catalase, GPx, glutathione reductase) and GSH levels [242,243,244,245,251,252], probably due to their free radical scavenging, tissue regenerative, and immune-stimulatory effects [5,251,252]. Multiple cooperative and synergistic mechanisms of propolis and its polyphenolic compounds are likely involved in protecting the whole organism from radiation. Propolis also boosted IgM production and increased CD8- and CD4-positive cells by 81%. It activated macrophages to secrete production cytokines such as IL-1, IL-8, IL-12, and TNF-α [5,251,252], with IL-1 and IL-12 promoting helper T-cell proliferation. In addition, propolis-activated macrophages released IFN-γ, which stimulated secondary activation of T-lymphocytes, leading to a reduction in IgG and IgM production. Macrophage-released cytokines after propolis treatment stimulated the proliferation of cytotoxic T-cells, indicating the activation of the cell-mediated immune response [5,251,252].

The differential radioprotection of normal tissues and radiosensitization of malignant cells is related to specific variations between normal and tumor cells in terms of metabolic, biochemical, and physiological signaling pathways, as well as environmental conditions, cellular communication, adhesion, and extracellular matrix (ECM)-receptor interactions. These factors include (i) inhibition of enzymes involved in DNA repair and expression; (ii) generation of ROS that cause DNA damage; (iii) the inability of tumor cells to effectively use additional antioxidants for repair; (iv) differences in cellular biochemistry or insufficient concentrations of flavonoids in tumor tissue to provide radioprotection; (v) the biodistribution of radioprotective compounds in both tumor and normal tissues, the hypoxic environment of tumors, and their poor vasculature; (vi) differences in the physiological and biochemical status of tumor versus normal cells at the time of irradiation; and (vii) the selective protection of normal tissues from radiation-induced damage while maintaining cytotoxicity to tumor cells [40,41,251,252].

### 4.3. Mechanisms of Radiotherapy Resistance of Cancer Cells

Radiotherapy resistance refers to a decrease in the effectiveness of radiation as an antitumor therapy. Similar to chemotherapy resistance, radiotherapy resistance can develop through various mechanisms, including tumor heterogeneity, changes in the surrounding microenvironment, and numerous gene alterations. In general, cancer is a diverse set of diseases, characterized by genetic and epigenetic deregulations, resulting in heterogeneous intra-tumoral cell phenotypes. Radiotherapy resistance can arise from either intrinsic factors, where cancer cells adapt their genes or phenotypes in response to radiation, or extrinsic factors, where the tumor microenvironment protects cancer cells from treatment.

Key contributors to radioresistance include the hypoxic tumor microenvironment and the modulation of mitochondrial and glycolytic pathways, Keap1/Nrf2-related mechanisms, as well as homologous recombination and non-homologous end joining in DNA repair. Hypoxia, together with a microenvironment composed of extracellular matrix, blood vessels, signaling molecules, and non-malignant cells like stromal cells, fibroblasts, and immune cells (such as M2 macrophages, N2 neutrophils, and myeloid-derived suppressor cells), plays a critical role in (i) increasing malignancy potential [1,5,13,14,117,182,286,287,288], (ii) promoting resistance to chemotherapy, immunotherapy, and radiation [286,287,288], and (iii) enhancing EMT and metastasis, leading to poor prognosis [288].

Radiotherapy resistance is associated with cancer recurrence, poor treatment responses and prognosis, and shorter patient survival, increasing both the cost and complexity of treatment [286,287,288]. In addition, radiotherapy resistance affects surrounding healthy tissues, resulting in side effects like dermatitis, radiation-induced diarrhea, rectal bleeding, anomia, infections, and a heightened risk of secondary cancers or chronic inflammatory diseases, including type II diabetes, cardiovascular diseases, brain dysfunction, cataracts, and immune system decline [286,287,288]. Chronic inflammation and ROS further complicate treatment, as they can suppress T-cells and NK cells while promoting the recruitment and polarization of M2 macrophages. This allows cancer cells to evade immune detection. Repeated radiation exposure can also stimulate the production of pro-survival factors, such as NF-κB, Akt, MMP-2, cell cycle proteins, and pro-inflammatory cytokines, all of which contribute to increased radiation resistance. Meanwhile, ROS and inflammation drive tumor invasion and metastasis by promoting EMT in cancer cells [1,5,286,287,288].

Even with improvements in radiotherapy protocols, tumor regrowth and relapse often occur due to either intrinsic or acquired resistance mechanisms. These mechanisms include enhanced DNA repair, adaptation to hypoxia, increased cell proliferation and survival, EMT, inhibition of apoptosis, and the activation of autophagy. HIF-1 also mediates resistance by inducing drug efflux transporters like MDR1/P-gp, repairing DNA damage, reprogramming tumor metabolism, reprogramming tumor metabolism, and blocking apoptosis while enhancing autophagy [286].

There are two subtypes of hypoxia in solid tumors: acute and chronic [286,289]. Acute hypoxia, caused by impaired blood flow, results from the transient narrowing or occlusion of blood vessels, limiting oxygen supply [290]. In contrast, chronic hypoxia, or diffusion-limited hypoxia, arises when oxygen diffusion from tumor blood microvessels to surrounding tissues is severely restricted [290]. Anemic hypoxia occurs when oxygen transport is reduced by chemotherapy [291,292]. Although the biological effects of acute and chronic hypoxia are debated, some studies suggest that acute hypoxia leads to a more aggressive tumor phenotype and increased metastatic potential [289].

**Figure 5:**
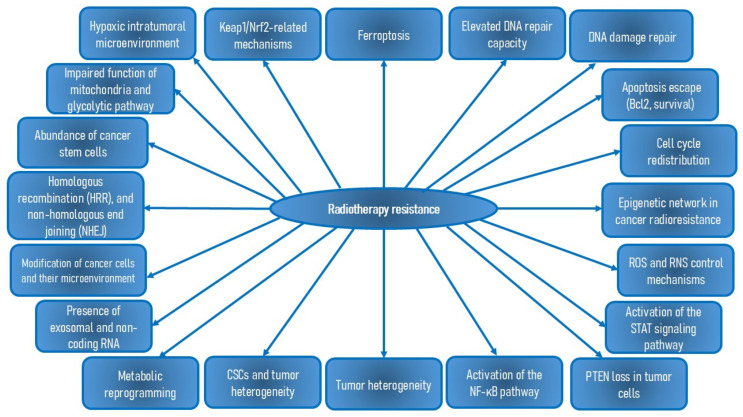
Molecular mechanisms of cancer radioresistance. Hypoxia plays a central role in regulating tumor growth and significantly contributes to increased resistance to radiation therapy (RT) by two to three times. Under hypoxic conditions, hypoxia-inducible factors (HIFs) are activated, triggering signaling pathways involved in epithelial–mesenchymal transition (EMT), which promotes EMT and enhances tumor resistance. HIFs also stimulate the secretion of vascular endothelial growth factor (VEGF) from tumor cells, supporting tumor vascular regeneration, offering protection to vascular endothelial cells, and counteracting the cytotoxic effects of radiation. Furthermore, HIF-1 activation influences key glycolytic enzymes, leading to the production of NADPH and glutathione, which neutralize post-irradiation reactive oxygen species (ROS) and reduce DNA damage. This also results in the excretion of large amounts of lactate, which contributes to radioresistance by activating myeloid-derived suppressor immune cells (MDSCs) in the tumor microenvironment through the GPR81/mTOR/HIF-1/STAT3 pathway. Hypoxia additionally sustains the stem cell-like properties of cancer stem cells (CSCs) that are inherently resistant to radiation. CSCs primarily function by reducing radiation-induced DNA damage and enhancing DNA repair mechanisms. Moreover, aberrant activation of Nrf2, often due to Keap1 or Nrf2 mutations or other Keap1/Nrf2-related mechanisms, further increases cancer cell resistance. In many cancer cells, hypermethylation of the Keap1 promoter leads to reduced Keap1 expression, disrupting the Nrf2-Keap1 pathway and contributing to both radioresistance and chemoresistance. Abbreviations: Keap/Nrf2, Kelch-like ECH-associated protein 1; nuclear factor erythroid-derived 2-like 2; NF-κB, nuclear factor kappa-light-chain-enhancer of activated B cells; Bcl-2, B-cell lymphoma 2; CSCs, cancer stem cells; STAT1, signal transducer and activator of transcription 1; ROS, reactive oxygen species; RNS, reactive nitrogen species; PTEN, phosphatase and tensin homolog.

Radioresistance is a major contributor to cancer progression and mortality, making it a significant challenge in the treatment of locally advanced, recurrent, and metastatic cancers. Cancer cells develop resistance to radiotherapy through several mechanisms, including (i) hypoxic intratumoral microenvironment; (ii) dysfunctional mitochondria and altered glycolytic pathways; (iii) Keap1/Nrf2-related mechanisms (disruption of the Keap1/Nrf2 pathway, such as Keap1 or Nrf2 somatic mutations or Keap1 promoter hypermethylation); (iv) enhanced DSBs DNA repair via homologous recombination and non-homologous end-joining pathways; (v) tumor heterogeneity; (vi) efficient SSBs DNA damage repair through ATR/Chk1 pathway and PARP1; (vii) evasion of apoptosis; (viii) cell cycle redistribution; (ix) an abundance of CSCs; (x) modifications of cancer cells and their microenvironment; (xi) the presence of exosomal and non-coding RNA; (xii) metabolic reprogramming; (xiii) resistance to ferroptosis; and (xiv) epigenetic alterations contributing to radioresistance (see Figure 5). Additional factors that influence radioresistance include the local tumor microenvironment, membrane signal sensors, the patient’s immune system, gut microbiome, diet, and mental health.

Radioresistance occurs through reduced ROS generation in irradiated cells, leading to diminished DNA damage (“oxygen effect”). Hypoxic cells are approximately 2.5 to 3 times more resistant to radiation than normal cells. As in dormant CSCs, DNA damage repair mainly occurs via the non-homologous end-joining pathway; the inhibition of this pathway is a potential strategy for overcoming CSC-associated radioresistance [1,5,49,293]. Although CSCs are the primary source of resistance and a target of epigenetic changes, targeting the epigenetic network may enhance the effectiveness and specificity of radiotherapy [293]. In addition to epigenetic alterations, tumor heterogeneity significantly contributes to radiation resistance [286,287,288].

There is a growing interest in developing therapies that increase the radiosensitivity of cancer cells while reducing systemic toxicity, thus improving patient outcomes. One of the major challenges with radiosensitizers is the possibility of increased damage to normal tissues. Therefore, the focus is on finding radiosensitizers that lower the radiation dose threshold for cancer cells without enhancing normal tissue radiosensitivity [251,252,255,294,295,296,297].

According to Huang and Zhou [298] and Jain et al. [299], two key factors are essential for improving resistance: (i) identifying regulators that contribute to the development and progression of radioresistance and (ii) finding effective strategies to overcome resistance induced by these regulators. Their work describes in detail how radiation-induced DNA damage triggers cellular responses. Radiation causes various direct DNA damage, including DNA cross-links, single-strand breaks, double-strand breaks, and DNA-protein cross-links. Among these, double-strand breaks are the most lethal, as they activate the DNA damage response, which includes DNA damage sensors, early signaling pathways, cell cycle arrest, and repair mechanisms. Several DNA damage response-related proteins, such as DNA-PKcs (DNA-dependent protein kinase, catalytic subunit), ATM/ATR (ataxia-telangiectasia mutated and Rad3-related), the MRN complex, PARP family proteins, MDC1, Wee1, LIG4 (ligase IV), CDK1, BRCA1, CHK1, and HIF-1 are potential targets for radiosensitization and could be used to overcome tumor radioresistance [298].

### 4.4. Propolis and Its Polyphenolic/Flavonoid Components as Radiosensitizers Can Overcome Tumor Resistance to Radiotherapy

Radiosensitizers can be classified into five main categories: (i) agents that suppress intracellular thiols or other endogenous radioprotective substances, (ii) substances that generate cytotoxic products through radiolysis, (iii) inhibitors of biomolecule repair such as inhibitors targeting DNA damage response (DDR) proteins, (iv) thymine analogs that can incorporate into DNA, and (v) oxygen mimics with electrophilic activity [300]. Certain radiosensitizers may enhance the radiation response by prolonging DNA damage and disrupting repair mechanisms.

Radiation can trigger hypoxia, fibrosis, vascular damage, chronic inflammation, and immunosuppression within the tumor microenvironment, all of which contribute to radiotherapy resistance. Propolis and its components have been shown to modulate several mechanisms involved in overcoming resistance, including reducing inflammation and mitigating tumor hypoxia by stabilizing p53 through its interactions with HIF1-α under hypoxic conditions [5,117]. This, in turn, reduces the activity of vasculogenic cells and inhibits vascular growth. Flavonoids are capable of detecting and destroying genetically mutated cells, decreasing the expression of antiapoptotic proteins like Bcl-2, and increasing pro-apoptotic proteins such as Bax, caspase 3, and procaspase 9 in cancer cells [5,6,7,8,9,117]. Propolis treatment also elevated the levels of cleaved PARP, decreased the expression of uncleaved PARP, and acted as a HDAC inhibitor (see review paper [5]). Furthermore, propolis and its flavonoid components can induce radiosensitization by targeting cancer stem cells (CSCs), resistant to radio- and chemotherapy with unlimited regenerative properties, and by regulating their self-renewal through the inhibition of key survival signaling pathways [1,5,49,203].

Several flavonoids have been reported to enhance the radiation response by prolonging DNA damage and impairing repair mechanisms. Some examples include genistein, quercetin, flavopiridol, myricetin, apigenin, and vicenin-2 [235,300,301,302,303,304]. Many natural flavonoids, including quercetin, chrysin, baicalein, apigenin, lutein, and kaempferol, as well as their derivatives, act as inhibitors of HIF-1, regulating main glycolytic enzymes such as pyruvate kinase M2 (PKM2), lactate dehydrogenase (LDH), glucose transporters (GLUTs), hexokinase II (HKII), phosphofructokinase-1 (PFK-1), and pyruvate dehydrogenase kinase (PDK) in various cancer cells [5,305,306,307,308,309,310,311,312,313,314,315,316,317,318,319,320,321,322,323,324,325]. For example, quercetin inhibited HIF-1α accumulation and synthesis under hypoxic conditions in several cancer cell lines, including LNCaP prostate cancer cells, SkBr3 breast cancer cells, and CX-1 colon cancer cells (5,312,317,320). Moreover, quercetin increases the therapeutic index of chemotherapy and radiotherapy by differentially affecting HIF-1α levels in healthy and cancer cells. In tumor cells like 4T1, quercetin reduced intra-tumoral HIF-1α in a hypoxia-dependent manner, while in healthy cells, it increased HIF-1α accumulation [310,311]. Chrysin, a compound found in propolis and honey, increased HIF-1α prolyl-hydroxylation, leading to its degradation. Chrysin also disrupted HSP90/HIF-1α binding, reducing HIF-1α stability in DU145 human prostate cancer cells, and modulated HIF-1α expression through the PI3K/Akt pathway [5,61,82,303,305].

Apigenin inhibits cancer cell proliferation and angiogenesis while significantly reducing the mRNA and protein levels of HIF-1α, VEGF, and GLUT1 under both normoxic and hypoxic conditions in pancreatic cancer cell lines (S2-013 and CD18). On the other hand, luteolin suppresses HIF-1 activity in M2-like TAMs under hypoxic conditions [5,306]). It is known that radiation-induced ROS can inhibit T-cells and NK cells while promoting the recruitment and polarization of M2 macrophages, enabling cancer cells to escape immune detection. HIF-1 inhibition promotes the transcription of EMT-related factors such as Snail, ZEB1, TWIST, and TCF3, all of which repress E-cadherin expression [5,306]. HIF-1 also indirectly contributes to EMT through other pathways, such as chemokine, NF-κB, TGF, and Notch signaling pathways, but all of them can be inhibited by flavonoids [5,306]

CAPE has been shown to act as a radiosensitizer in certain cancers, including breast cancer, where estrogen receptor signaling is crucial for cell proliferation and survival [307]. Khoram et al. [307] investigated whether CAPE could enhance the radiation sensitivity of two cell lines: MDA-MB-231 (estrogen receptor-negative; lacks ERα and has negligible expression of ERβ) and T47D (estrogen receptor-positive; expresses both ER α and β). Due to CAPE’s structural similarity to estrogen, it acts as an estrogen modulator, showing a higher affinity for ERβ than ERα. As a result, CAPE caused more severe damage in T47D cells. This damage included double-strand breaks, which are highly lethal. CAPE also delays DNA repair, with T47D cells showing repair delays of up to 120 min, compared to 60 min in MDA-MB-231 cells after irradiation. CAPE prevents DNA repair by inhibiting the NF-κB pathway, thereby increasing radiosensitivity [308].

Van Rijn’ et al. [254] demonstrated that flavonoids such as genistein, apigenin, and quercetin, when applied post-irradiation to hepatoma cells, enhanced cell death. These compounds appeared to exert multiple effects, including inhibition of PDGF- and EGF-induced mitogenesis and DNA synthesis in both liver and fibroblast cells. The reduction in cell survival was attributed to the inhibition of protein tyrosine kinases (PTKs) and decreased repair of DNA radiation damage, which slowed down cell repopulation, possibly through the inhibition of topoisomerase II activity. The radiosensitizing effects of quercetin were further tested in tumor cell lines such as DLD1, HeLa, and MCF-7 by colony formation assays [255,257]. Lin et al. demonstrated that a combination of quercetin with radiotherapy can enhance tumor radiosensitivity by targeting the ATM-mediated pathway in response to radiation in tumor cell lines and in human colorectal cancer xenograft models in nude mice. The ability of quercetin to enhance radiosensitivity has also been investigated in brain tumors such as glioblastoma, glioma, neuroblastoma, astrocytoma, and medulloblastoma [310]. The authors point out that quercetin has the capacity to modulate intricate signaling pathways, trigger apoptosis, impede cell migration, and enhance radiosensitivity in brain tumor cells. In addition, pre-treatment with quercetin increased the radiosensitivity of colon cancer cells (HT-29 and DLD-1) by targeting the Notch-1 pathway and CSCs [311].

Genistein has been found to selectively enhance radiosensitivity in A549 cells by inhibiting the methylation of the Keap1 gene promoter and inducing apoptosis and autophagy via Beclin-1 while suppressing Bcl-xL and its interactions with Beclin-1. When combined with the tyrosine kinase inhibitor AG1024 (tyrphostin), genistein further increased the radiosensitivity of prostate cancer cells by suppressing the homologous recombination and non-homologous end-joining pathways [312,313]. Similarly, it has been reported that a combination of genistein and radiation increases radiosensitivity in human esophageal and cervical cancer cells and inhibits DNA synthesis, cell growth, and colony formation [314].

Radiation also activates pathways PI3K/Akt, ERK, and NF-κB, which contribute to resistance by upregulating anti-apoptotic proteins and proteins involved in DNA repair [266,315,316]. Flavonoids, in combination with radiation, have been shown to inhibit these pathways, reducing cancer cell survival, inducing cell cycle arrest, and promoting apoptosis [266,315,316]. The reduced ability of cancer cells to form colonies in the combination of flavonoids (genistein, quercetin, flavopiridol, myricetin, apigenin and vicenin-2) with radiation shows that flavonoids contribute to the sensitization of cancer cells through various mechanisms including cell cycle arrest, increased pro-oxidative effect leading to increased DNA damage and downregulation of DNA repair pathways and consequently stimulation of apoptotic proteins and suppress antiapoptotic proteins [316]. Flavonoids also inhibit the PI3K/Akt, ERK, and NF-κB pathways, which are upregulated with radiation and promote apoptosis. The specificity for flavonoid action can be attributed to the ability of flavonoids to undergo oxidation with metals, resulting in the formation of free radicals together with H_2_O_2_, leading to an increase in flavonoid-induced cancer cell apoptosis [316]. For example, cancer cells have 20% higher levels of Cu ions due to the increased requirement of Cu by the proteins involved in cell proliferation and angiogenesis. Phenolic compounds, via a copper–redox reaction, induce the production of reactive oxygen and electrophilic intermediates, causing a spectrum of lesions in DNA. Increased copper levels in tumor cells contribute to cytotoxicity and tumor cell death and could be a selective mechanism for killing tumor cells [110]. For example, curcumin shows an anti-inflammatory effect by inhibiting NF-κB, a key player in both tumorigenesis and radioresistance. In prostate cancer cells (PC-3), radiation-induced TNF-α increased NF-κB activity, resulting in the induction of Bcl-2 protein. However, curcumin combined with radiation prevented TNF-α-mediated NF-κB activity, resulting in Bcl-2 downregulation. Moreover, this combination activated cytochrome c and caspase-9 and -3, further promoting apoptosis. Curcumin has thus been identified as a potent radiosensitizer in prostate cancer by overcoming radiation-induced pro-survival gene expression [317,318].

Liao et al. [319] found that resveratrol enhances radiosensitivity in human NSCLC NCI-H838 cells by inhibiting NF-κB activation. Similarly, Da Costa [320] demonstrated that resveratrol, in combination with 3 Gy ionizing radiation, induces significant cytotoxicity and antiproliferative effects in MCF-7 breast cancer cells. This was associated with necrosis, senescence, and extrinsic apoptosis via a decrease in the Bax/Bcl-2 ratio and increased caspase-8 activity. In addition, resveratrol disrupted oxidative metabolism, increasing oxidative damage of proteins, lipids, and membranes and decreased antioxidant enzyme activity in cancer cells. In combination with radiation, flavonoids exert pro-oxidant effects, exacerbating DNA damage and increasing ROS, ultimately promoting apoptosis. Thus, resveratrol demonstrated pro-oxidant effects in MCF-7 and ovarian cancer cells [320]. Resveratrol also enhanced radiosensitivity in nasopharyngeal carcinoma cells by downregulating E2F1 [321]. Flavonoids, in general, were found to inhibit DNA repair pathways [322,323,324,325].

**Figure 6:**
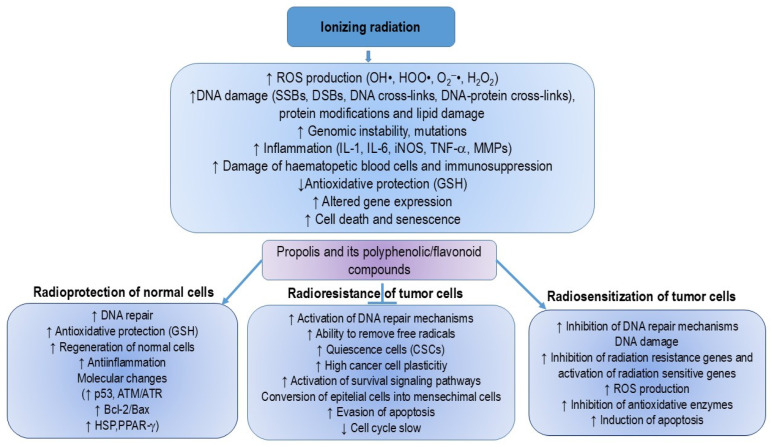
Physical, biochemical, and biological consequences of radiation and potential mechanisms by which propolis and its flavonoid components contribute to radioprotection, combat radioresistance, and enhance radiosensitivity of tumor cells. Radiation damages macromolecules like DNA and RNA, leading to single-strand breaks (SSBs) and double-strand breaks (DSBs), the latter being the most lethal. These damages impair genomic stability, cause mutations, and can lead to cell death or diseases such as cancer. Radiation also affects DNA bases, disrupts DNA-DNA and DNA-protein interactions, and can impair cellular and organ function. This damage triggers highly coordinated pathways, including the DNA damage response (DRR) and repair mechanisms. In the tumor microenvironment, radiation can induce hypoxia, fibrosis, vascular damage, chronic inflammation, and immunosuppression, all of which may contribute to radiotherapy resistance. Propolis and its components counteract these effects through several mechanisms, such as modulating inflammation, overcoming hypoxia by stabilizing p53 via its interaction with hypoxia-inducible factor-1 (HIF1-α), and reducing vasculogenic cell function and vascular growth. Flavonoids in propolis detect and eliminate genetically mutated cells, reduce the expression of antiapoptotic proteins (e.g., Bcl-2), and increase pro-apoptotic proteins (e.g., Bax) in cancer cells. Propolis and its flavonoids also sensitize cancer cells to radiation by targeting radiation-activated pathways, including receptor tyrosine kinases (RTKs), NF-κB signaling, DNA damage response, cell cycle regulation, and apoptosis. The radioprotective effects of propolis and its polyphenolic flavonoids involve (i) antioxidant properties that neutralize free radicals, reduce oxidative stress, and prevent further cellular damage; (ii) stimulation of DNA repair, increasing cell survival; (iii) anti-inflammatory effects that mitigate radiation-induced inflammation and tissue damage; and (iv) modulation of the immune system, supporting recovery from radiation-induced damage. Propolis also acts as an anti-inflammatory agent by targeting key components in the inflammatory response, including the inhibition of Toll-like receptor 4 (TLR4), myeloid differentiation primary response 88 (MyD88), interleukin-1 receptor-associated kinase 4 (IRAK4), Toll/interleukin-1 receptor domain-containing adapter-inducing interferon-β (TRIF), nucleotide-binding oligomerization domain-like receptor protein (NLRP) inflammasomes, and NF-κB. Flavonoids in propolis regulate the tumor microenvironment, epithelial–mesenchymal transition, growth factor receptors, and pathways such as PI3K/Akt, MAP kinase/ERK, and Janus kinase in cancer cells. They also modulate NF-κB signaling, regulating ATP-binding cassette transporters, apoptosis, autophagy, cell cycle, and cancer stem cell activity, as well as oncogenes and gene repair control mechanisms. Abbreviations: ROS, reactive oxygen species, SSBs, single-strand breaks, DSBs, double-strand breaks IL-1, interleukin-1, IL-6, interleukin-6; iNOS, inducible nitric oxide synthase; TNF-α, tumor necrosis factor alpha; MMPs, matrix metalloproteinases; GSH, glutathione; p53, tumor suppressor protein p53; Bcl-2, B-cell lymphoma 2 protein; Bax, pro-apoptotic protein; ATM, ataxia-telangiectasia mutated kinase; ATR, Rad3-related (ATR) kinases; HSP, heat-shock protein; PPAR-γ, proliferator-activated receptor gamma; CSCs, cancer stem cells; ↑ upregulated; ↓ downregulated.

The critical aspect of cellular survival is the ability to accurately replicate and repair DNA while minimizing mutations. This requires a balanced supply of deoxyribonucleoside triphosphates (dNTPs), the building blocks of DNA synthesis. Ribonucleotide reductase plays a key role in this process by converting ribonucleotides to 2′-deoxyribonucleotides, providing the necessary precursors for DNA synthesis and repair [326]. It has been shown that propolis interferes with ribonucleotide reductase function, thereby increasing the radiosensitivity of breast cancer cells [327]. By acting as a ribonucleotide reductase inhibitor, propolis impairs DNA repair, leading to cell cycle arrest in the G1 phase and preferentially targeting cells in the S phase. Additionally, propolis exerts anti-inflammatory effects by downregulating important signaling proteins such as cooperation between TLR4, NLRP inflammasome, and NF-κB signaling pathways. For instance, by targeting NF-κB, flavonoid components found in propolis modify the tumor microenvironment and EMT, regulate growth factor receptors, and modulate key pathways like PI3K/Akt, MAPK/ERK, and Janus kinase in cancer cells [5,37,328]. Additionally, flavonoid-mediated modulation of NF-κB signaling affects ATP-binding cassette transporters, apoptosis, autophagy, and cell cycle and influences CSC activity, oncogenes, and gene repair mechanisms. Flavonoids that inhibit tumor growth by modulating NF-κB signaling include nobiletin, luteolin, icariin, EGCG, genistein, wogonin, and others [328]. Figure 6 illustrates the biochemical, physical, biochemical, and biological consequences of radiation, as well as the potential mechanisms through which propolis and its polyphenolic flavonoids contribute to radioprotection, overcome radioresistance, and enhance radiosensitization of tumor cells.

## 5. NF-κB as a Target in Overcoming Chemoresistance and Radioresistance

The activation of NF-κB plays a central role in the body’s defense mechanisms against infection and stress, acting as a key mediator of inflammatory responses. Normally, an acute inflammatory response is triggered rapidly and is autoregulated. However, chronic inflammation, a hallmark of cancer, is widely regarded as a critical factor in the onset and progression of various cancers. NF-κB provides a mechanistic link between inflammation, cancer development, and tumor resistance to radiation therapy (Figure 1 and Figure 3). NF-κB influences these processes both directly and indirectly by inducing genes responsible for inflammation, cancer cell proliferation and survival, EMT, invasion, angiogenesis, metastasis, genetic and epigenetic changes, CSC formation, cellular metabolism, and resistance to therapy. At the same time, it counteracts growth regulators, such as tumor suppressor p53 [5,37,329,330,331,332]. As a major regulator of apoptosis and cell cycle, p53 is directly and indirectly regulated by NF-κB [319].

NF-κB binds to specific promoter regions and regulates the transcription of more than 400 genes, including COX-2, cyclin D1, Bcl-2, Bcl-xL, survivin, and X-linked inhibitor of apoptosis protein (XIAP), which are involved in chemoresistance and radioresistance. NF-κB activation also promotes immunosuppression by increasing the production of IL-10 and COX-2 while inhibiting STAT1-dependent genes like NOS2, MHC II, and IL-12 [329]. Once activated by various cytokines, NF-κB further stimulates the production of additional pro-inflammatory cytokines, chemokines, adhesion molecules, acute phase proteins, inducible enzymes, and regulators of cell proliferation and apoptosis. NF-κB has been shown to play a crucial role in inhibiting apoptosis triggered by factors such as TNF-α, chemotherapy, and γ-radiation [330,331,332]. Tumor cells usually have high levels of constitutive NF-κB activity [5,328,329,330,331,332], and exposure to cytotoxic agents, including ionizing radiation, further enhances NF-κB activity [331], providing tumor cells with survival advantages and increased resistance to ionizing radiation and other cytotoxic treatments.

Pre-clinical models have shown that many chemotherapeutic agents, such as platinum-based drugs, anthracyclines, and taxanes, activate the NF-κB pathway [328,333,334,335,336]. Cancer cells lacking NF-κB activity show increased sensitivity to TNF-α and chemotherapeutic agents [333,337], making NF-κB a promising target for cancer therapy. Inhibiting the NF-κB signaling pathway could sensitize resistant cancer cells to chemotherapy and/or radiotherapy [330,331,332].

Additionally, studies have indicated that the resistance of human cervical carcinoma cells to cisplatin or doxorubicin is partially mediated by enhanced NF-κB activation [335,336]. Many researchers have found that inhibiting NF-κB potentiates the anticancer effects of chemotherapeutic drugs [333,334,335,336,337]. Our findings indicate that treatment with doxorubicin or cisplatin inhibits cancer cell growth and induces apoptosis [45,52,84,85,86,87,88,89,90,91,336,337,338,339]. However, pretreatment of cancer cells with propolis followed by cisplatin or doxorubicin significantly enhances cancer cell growth inhibition, possibly due to the induction of apoptosis and NF-κB inactivation by flavonoids, as proposed by Li et al. [338]. Li and colleagues demonstrated that flavonoids administered to mice prior to chemotherapy enhanced the antiproliferative and proapoptotic effects of these drugs. Both cisplatin and doxorubicin have been shown to induce significant apoptosis in various cancer cell types [339,340,341]. It is well established that proteins like Bcl-2, Bcl-xL, and survivin protect cells from apoptosis, whereas p21^WAF1^ suppresses cell growth and promotes apoptosis [328,329,330,331]. Li et al. [338] and Sharifi-Rad et al. [342] reported that genistein downregulates NF-κB, Bcl-2, Bcl-xL, and survivin while upregulating p21^WAF1^ [342,343]. As NF-κB binding sites have been identified in the promoters of Bcl-2, Bcl-xL, and survivin [45,338,339,340,341,342,343], it is suggested that genistein may inhibit their expression through NF-κB downregulation, thereby sensitizing cancer cells to apoptosis induced by docetaxel, cisplatin, or doxorubicin. However, other mechanisms may also contribute to this chemosensitization effect induced by genistein.

Moreover, Lee et al. [344] demonstrated the selective effects of genistein on the toxicity of bleomycin in normal lymphocytes and HL-60 cells, revealing dual, opposing actions. Genistein enhanced bleomycin-induced cytotoxicity in the human leukemia cell line HL-60 while protecting normal lymphocytes from bleomycin’s toxic effects. Guo et al. [345] suggested that isoflavonoids may exert antioxidant activity by inducing endogenous antioxidants like GSH, which could explain why genistein protected normal lymphocytes but not HL-60 cells. If genistein had direct scavenging ability, protection in both cell types would be expected [344,345].

Genistein has been shown to inhibit NF-κB activity and suppress the growth of various cancer cells without causing systemic toxicity. Li and colleagues [90,91] investigated whether NF-κB inactivation with genistein prior to chemotherapy could enhance cancer cell killing. In vitro and animal studies involving gene transfection revealed that genistein pretreatment enhanced the tumoricidal effects of chemotherapeutic agents like cisplatin and docetaxel in prostate (PC-3), breast (MDA-MB-231), lung (H460), and pancreatic (BxPC-3) cancer cells. Cells were pretreated with 15 to 30 µmol/L of genistein for 24 h, followed by exposure to low doses of chemotherapeutic agents for an additional 48 to 72 h [346,347]. Genistein pretreatment completely abolished the chemotherapy-induced increase in NF-κB activity observed within 2 h of cisplatin or docetaxel treatment. This result was confirmed in animal models, which showed that NF-κB was specifically targeted in vivo, as demonstrated by p65 cDNA transfection and small interfering RNA studies. Collectively, these findings suggest that genistein’s ability to inactivate NF-κB enhances cancer cell growth inhibition and apoptosis induced by cisplatin, docetaxel, and doxorubicin in prostate, breast, lung, and pancreatic cancers. CAPE, derived from propolis, has been identified as an effective anti-tumor agent in human prostate cancer cells, both androgen-dependent and androgen-independent [348]. CAPE inhibits mucosa-associated lymphoid tissue lymphoma translocation protein 1 (MALT1) expression, reducing cell proliferation, invasion, and tumor growth through modulation of the p53 and NF-κB signaling pathways. Combined treatment of docetaxel with CAPE inhibited the proliferation and survival of docetaxel-resistant prostate cancer cells via inhibiting Bcl-2 and c-Myc and induced metabolic interference and apoptosis.

Kubatka et al. proposed that flavonoids are important regulators of NF-κB signaling and negatively influence the fundamental cellular processes responsible for acquired cell plasticity and drug resistance [44]. Flavonoids, such as genistein, hesperidin, naringenin, flavokawain A, icariin, baicalein, wogonin, apigenin, CAPE, resveratrol, EGCG, luteolin, fisetin, norwogonin, methyl gallate, catechin-3-O-gallate, ursolic acid, and betulinic acid, and kaempferol, have been shown to resensitize tumor cells to chemotherapy by interfering with the NF-κB signaling pathway. This includes breast cancer cells, where they increase sensitivity to chemotherapeutic agents [15,44,49,60,66,178,181,182,183,320,321,322,323,324,325,326,327,333].

To maximize the effectiveness of cancer treatment, combining two or more chemotherapeutic agents is a common approach, but this may result in increased systemic toxicity [49,341,349,350]. Therefore, optimizing combination therapies with natural antioxidants based on molecular mechanisms could improve therapeutic outcomes. Such strategies are urgently needed for the treatment of cancer patients (Figure 1 and Figure 3).

Many cancer cells are resistant to ionizing radiation-induced cell death. Recently, it was reported that Ki-Ras is responsible for increased radioresistance [351], with K-ras mutations being particularly common in pancreatic, colon, and lung adenocarcinomas among the three Ras family genes (K-ras, N-ras, and Ha-ras). Kim et al. [351] found that inhibiting ionizing radiation-induced NF-κB activation, but not Akt or MAPK kinase (MEK), increased radiosensitization in Ki-Ras- transformed human prostate epithelial 267B1/K-ras cells. NF-κB activation also plays an antiapoptotic role in human leukemic K562 cells exposed to ionizing radiation [352,353]. In addition, constitutive NF-κB activity, basal apoptosis levels, and radiosensitivity have been reported in head and neck carcinoma cells [354,355,356]. Similar findings have been observed in other cancers, including breast and colon, further supporting the role of NF-κB in oncogenesis and resistance to cell death [44,356]. Given the overwhelming evidence that NF-κB plays a crucial role in tumor resistance to radiotherapy and chemotherapy, targeting NF-κB inhibition at the molecular level is being actively pursued as a novel adjuvant strategy in cancer treatment [357,358].

Many studies have reported that propolis and its flavonoids, including quercetin, chrysin, resveratrol, and CAPE, inhibit the growth of human breast cancer and other tumor cells by modulating the NF-κB signaling pathway [5,178,179,180,181,332,333,334,335].

**Figure 7:**
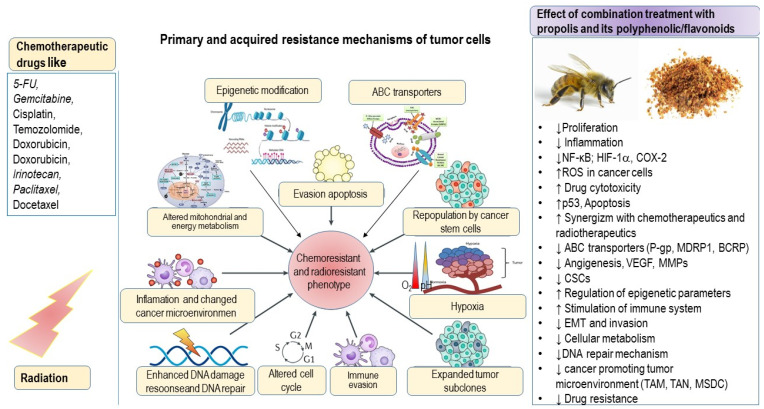
The effect of propolis and its components on resistance mechanisms induced by chemotherapy and radiotherapy. Propolis and its polyphenolic flavonoid components, by downregulation of NF-κB, HIF-1α, and COX-2, can inhibit cell proliferation, inflammation, invasion, metastasis, and angiogenesis and induce pro-apoptotic pathways. The inhibition of ABC transporters increases DNA damage, regulates MDR resistance, activates apoptotic cells, and regulates the expression of metabolic enzymes, as well as chemosensitivity in various types of cancer cells. Furthermore, by enhancing the expression and activity of p53, they downregulate antiapoptotic PI3K signaling and MAPKs to promote endogenous ROS generation and overexpression of antiapoptotic genes such as Bcl-2. Flavonoids have dual action regarding ROS homeostasis—they act as antioxidants under normal conditions and are potent pro-oxidants in cancer cells, triggering the apoptotic pathways and downregulating pro-inflammatory signaling pathways. Increased activation of p53 regulates numerous immune pathways, such as the major histocompatibility complex I (MHC-I) associated pathway, toll-like receptors (TLRs), and immune checkpoints. Flavonoids, by upregulating p53, can also target the tumor microenvironment by reprograming Tregs and TAMs, reducing angiogenesis, and decreasing the number of immunosuppressive cells or reactivating their anti-tumor functions. This includes reducing the expression of PD-1 and/or PD-L1 on T-cells. Reprogramming of the tumor microenvironment and reactivation of cells of the immune system inhibits the stemness of CSCs. Furthermore, inhibition of important signaling pathways, including STAT3, NOTCH, PI3K, WNT/β-catenin, and NANOG, also contributes to CSC stemness inhibition. Flavonoid components from propolis, as inhibitors of HIF-1, have the ability to regulate critical glycolytic components in cancer cells, including pyruvate kinase M2(PKM2), lactate dehydrogenase (LDHA), glucose transporters (GLUTs), hexokinase II (HKII), phosphofructokinase-1 (PFK-1), and pyruvate dehydrogenase kinase (PDK). Flavonoid components from propolis can reverse resistance to chemotherapeutics induced by hypoxia through the suppression of glycolysis and PTEN/Akt/HIF-1a signaling. The ability of flavonoids to enhance the antitumor activity of chemotherapeutics by inducing apoptosis and cell cycle arrest may be partially mediated through epigenetic regulation by inhibiting histone deacetylases (HDAC). By inhibiting HDAC activity, flavonoids enhance p53 acetylation, increasing the expression of p53 target genes involved in cell death and growth arrest. Abbreviations: NF-κB, nuclear factor kappa-light-chain-enhancer of activated B cells; CSCs, cancer stem cells; ROS, reactive oxygen species; STAT, signal transducer and activator of transcription, VEGF, vascular endothelial growth factor receptor; MDR, multi-drug resistance; ABC transporter, ATP-binding cassette (ABC) transporter proteins; P-gp, P-glycoprotein; MDRP1, multidrug resistance-associated protein 1; BCRP, breast cancer resistance protein; EMT, epithelial–mesenchymal transition; MMPs, matrix metalloproteinases; HIFs, hypoxia-inducible factors; COX-2, cyclooxygenase-2; TAM, tumor-associated macrophage; TAN, tumor-associated neutrophils; MSDC, myeloid-derived suppressor cells; Treg, regulatory T-cells; STAT3, signal transducer and activator of transcription 3; MAPK, mitogen-activated protein kinase; M1 cells, M1 macrophages; M2 cells; M2 macrophages; MHC-I, the major histocompatibility complex class I; ↑ upregulated; ↓ downregulated.

The potential role of propolis and its polyphenolic components in overcoming tumor cell resistance to chemotherapy, radiotherapy, and other cancer treatments lies in their ability to inhibit NF-κB. This inhibition not only reduces the negative effects of cancer and its treatments on the body but also strengthens the immune response. NF-κB is involved in several key processes in cancer progression: (i) stimulation of tumor cell proliferation (ii) regulation of angiogenesis, (iii) induction of EMT, (iv) dysregulation of apoptosis, autophagy, and the cell cycle, (v) promotion of chronic inflammation and creation of the pro-tumor microenvironment by suppressing M1 macrophages and inducing M2 polarization, (vi) evasion of immune cell recognition and destruction, (vii) activation of CSCs, (viii) development of distant metastases, (ix) resistance to chemotherapy and radiotherapy, and (x) reprogramming energy metabolism and weakening the immune system to support tumor growth. Possible mechanisms of overcoming drug resistance pathways using propolis and its polyphenols/flavonoids, along with chemotherapy and radiotherapy, are shown in Figure 7**.**

However, it should be noted that numerous signaling pathways are associated with inflammation, including NF-κB, AP-1, peroxisome proliferator-activated receptor (PPAR) and Nrf2 transcription factors; MAPKs; protein tyrosine kinases (PTKs); PI3K/Akt; and the ubiquitin–proteasome system [359].

NF-κB is found in immune cells such as macrophages, neutrophils, or lymphocytes, elements of the tumor microenvironment that possess the ability to generate highly reactive species involved in direct DNA damage, stimulation of carcinogenesis, angiogenesis and clonal growth of initiated cells and inhibition of apoptosis [147,360,361]. Contrary to the NF-κB pathway, the Nrf2 pathway is responsible for cytoprotection, and its activation leads to the expression of several cytoprotective enzymes and proteins involved in cellular defense against ROS and electrophilic species. The Nrf2 activation increases the expression of NAD(P)H: quinone oxidoreductase (NQO1), glutathione -S-transferases (GSTs), and anti-apoptotic proteins such as Bcl-2 and Bcl-xL, decreasing ROS levels and protecting the cell against oxidative stress [359,362]. In addition to the role of the Nrf2 pathway in maintaining cellular redox and electrophilic homeostasis, it has been shown that Nrf2 overexpression in cancer cells may contribute to increased proliferation, invasion, and chemoresistance. As mentioned, Nrf2 gene mutation together with epigenetic mechanisms, such as Nrf2 or Keap1 promoter methylation and microRNA, confers malignant potential and resistance to therapy. Thus, the overproduction of ROS contributes to MDR by upregulating pathways such as MAPK, NF-κB, PI3K/Akt/STAT3, and Keap1-Nrf2-ARE. Studies have confirmed that cells expressing Nrf2 are resistant to chemotherapeutic agents like doxorubicin, etoposide, and cisplatin due to increased production and the upregulation of MRP1 transporters. Nrf2, as a transcription factor, regulates genes responsible for the synthesis of endogenous antioxidants (e.g., HO-1), transporters (e.g., MRP1, MRP2), and detoxifying enzymes (e.g., glutathione-S-transferase) [363,364].

Since Nrf2 and NF-κB transcription factors are directly involved in many steps of carcinogenesis, their concomitant modulation, i.e., induction of Nrf2 and inhibition of NF-κB in normal cells and inhibition of both in cancer cells, may be considered the best strategy for cancer chemoprevention and therapy, respectively.

ROS, produced during oxidative stress, play a role in initiating and promoting carcinogenesis by activating or suppressing redox-sensitive transcription factors, of which Nrf2 is particularly interesting due to its antagonistic relationship with NF-κB. Nrf2 encodes genes responsible for antioxidant defense and cytoprotection, while NF-κB regulates the expression of pro-inflammatory genes. Nrf-2 activation triggers intracellular events that suppress NF-κB and vice versa. The MAPK family also contributes to the coordinated regulation of both Nrf2 and NF-κB.

Flavonoids have been shown to activate the Nrf2/ARE pathway even in the absence of oxidative stress. In normal cells, this activation can offer chemopreventive effects by inducing Nrf2/ARE-driven cytoprotective genes. However, in cancer cells, constitutive activation of the Nrf2/ARE promotes continuous proliferation, survival, and the renewal of CSCs, as well as resistance to chemotherapy. Flavonoids can exhibit a hormetic effect, meaning they can have both antioxidant and prooxidant actions depending on their concentration. Under normal physiological conditions, activation of the Nrf2/ARE pathway by endogenous or exogenous stimuli helps maintain redox homeostasis, contributing to the chemoprotective and radioprotective effects of flavonoids. At low concentrations (1.5 to 20 µM), flavonoids such as luteolin, apigenin, myricetin, quercetin, naringenin, epicatechin, genistein, and daidzein can promote cancer cell growth and proliferation in vitro, while others, such as luteolin, apigenin, and chrysin inhibit the Nrf2 /ARE pathway and have the opposite effect. Interestingly, luteolin can act as an inhibitor of both Nrf2 and NF-κB [359]. Quercetin induces apoptosis in vivo in human xenograft acute myeloid leukemia (AML) and in vitro in leukemia cell lines by reducing nuclear translocation of Nrf2, inducing proteasomal degradation of Nrf2 and upregulating the histone deacetylase HDAC4, which leads to upregulation of pro-apoptotic miRNAs. Quercetin can also induce apoptosis through its DNA methylation activity by inhibiting HDAC and modifying histones H3ac and H4ac, which enables the transcription of genes whose products are involved in the apoptosis pathway. In addition to quercetin, other polyphenol components, such as urosolic acid and resveratrol, can modify the Nrf2 pathway [5,19,365,366].

Given the pleiotropic role of NF-κB, inhibiting this pathway using propolis and its polyphenolic/flavonoid components in myeloid or tumor cells could lead to tumor regression, making NF-κB a promising therapeutic target. The cellular and molecular mechanisms of propolis and its polyphenolic/flavonoid components involved in tumor regression are discussed in detail in the review by Oršolić and Jazvinšćak Jembrek [5]. The interplay between the Nrf2 and NF-κB pathways as important regulators of the response to oxidative stress and inflammation in the body was described in [359,362,367]

**Figure 8:**
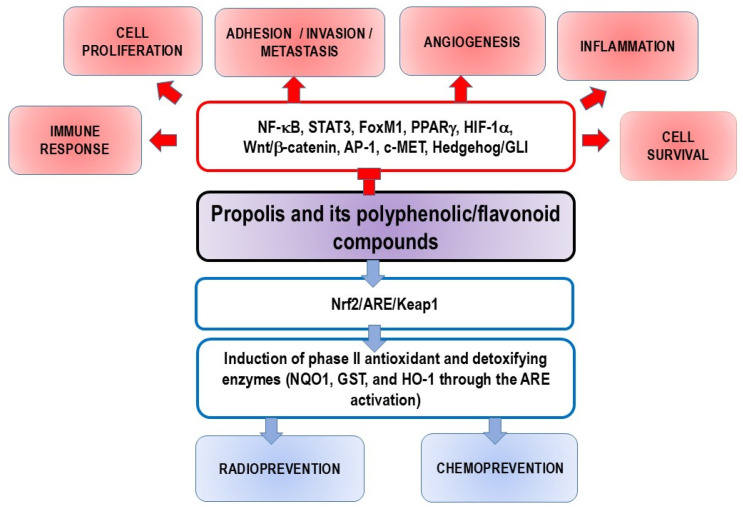
The interaction of propolis and its polyphenolic/flavonoid compounds with key signaling pathways involved in cancer pathogenesis and progression. Reactive oxygen species (ROS), generated by oxidative stress, contribute to the initiation, promotion, and malignant transformation of cancer by activating or suppressing redox-sensitive transcription factors. Nrf2 regulates genes responsible for antioxidant defense and general cytoprotection, while NF-κB controls the expression of pro-inflammatory genes. Nrf-2 activation triggers intracellular events that inhibit NF-κB and vice versa. The MAPK family plays a role in coordinating the modulation of Nrf2 and NF-κB pathways. Targeting NF-κB with flavonoids present in propolis involves altering the tumor microenvironment, EMT, regulation of growth factor receptors, and the modulation of pathways such as PI3K/Akt, MAPK/ERK, and Janus kinase/signal transduction in cancer cells. In addition, the modulation of NF-κB signaling by flavonoids affects ATP-binding cassette transporters, apoptosis, autophagy, and cell cycle and affects the activity of CSCs, oncogenes, and the regulation of gene repair.

In summary, inhibiting NF-κB with propolis and its flavonoids leads to several beneficial changes: (i) modification of the tumor microenvironment and suppression of EMT; (ii) regulation of growth factor receptors and modulation of key pathways like PI3K/Akt, MAPK/ERK and Janus kinase/signal transduction in cancer cells; (iii) control of ATP-binding cassette transporters; (iv) regulation of the cell cycle, apoptosis, and autophagy; (v) changes in CSC activity and oncogene regulation; and (vi) involvement in DNA repair processes. Notably, flavonoids can also regulate HIFs, the cell cycle, CSCs, autophagy, and critical enzymes such as STAT3, p53, and NF-κB, confirming their multi-functional properties, as summarized in Figure 8.

Abbreviations: NF-κB, nuclear factor kappa-light-chain-enhancer of activated B cells; STAT3, signal transducer and activator of transcription 3; FoxM1, forkhead box protein M1; HIF-1α, hypoxia-inducible factor 1-alpha; Wnt β catenin, Wnt/β-catenin pathway; PPARγ, peroxisome proliferator-activated receptor gamma; AP-1, activator protein 1; c-MET, tyrosine-protein kinase Met or hepatocyte growth factor receptor (HGF); Hedgehog/GLI, Hedgehog/GLI pathways; Nrf2, nuclear factor erythroid 2–related factor 2; ARE, antioxidant response element; NQO1, quinone oxidoreductase 1, Keap1, Kelch-like erythroid cell-derived protein 1; HO-1, heme oxygenase-1; GST, glutathione S-transferases; MAPK, mitogen-activated protein kinase

## 6. Low Bioavailability and Side Effect of Propolis and Its Polyphenolic/Flavonoid Components

Propolis has a variable and complex chemical composition with high concentrations of flavonoids and phenolic compounds present in the extract and largely depends on the geographical area, climatic conditions, the plants that live there, the period of propolis collection, as well as the type of bees. Different samples of propolis contain as main secondary metabolites of phenolic substances, especially flavonoids, belonging to different sub-classes such as flavanones, flavones, flavonols, and dihydroflavonols, which constitute more than 50% of the propolis weight [5,6,7,8,9]. Flavonoids are special components of propolis, which have been identified in propolis so far, and more than 150 species have been studied. These compounds, generally present in the plant kingdom as glycosides, are mostly present in propolis as aglycones, probably due to the action of bees’ enzymes (glucosidase) during harvesting and transport. After the ingestion of polyphenols into the organism per os and their metabolization by intestinal and liver enzymes as well as intestinal microflora, their metabolites are found in the bloodstream in a thousand times lower concentration than at entry. Large amounts of parent compounds and metabolites pass through the large intestine, where they are broken down by local microbes to less phenolic acid and aromatic catabolites, which then are absorbed into the bloodstream [368,369,370]. The bioavailability of propolis and its/polyphenolic components depends on many metabolic factors (glycosylation, esterification, and polymerization), which is still the subject of research. In addition to the above-mentioned factors, the complexity of the bioavailability of polyphenols is influenced by: (i) chemical structure, (ii) liberation from resinous secretions from flower and leaf buds, (iii) absorption in the gastrointestinal system, (iv) metabolism by gut microbiota, liver, enterocytes, (v) plasma transport, plasma concentration, and (vi) different distribution and elimination [368,369,370].

In the last few years, a large number of studies have appeared on the effectiveness of polyphenols on the brain, vascular system, neoplasms, and inflammation [5,49,69,70,71,72,73,74,75,371]. Their bioavailability and distribution in tissues are crucial for the effectiveness of flavonoids and health effectiveness [5,49,69,70,71,72,73,74,75,371]. However, the data so far point to numerous shortcomings, including insufficiently clear conclusions, the subjectivity of researchers, inadequate controls, the absence of a mechanism to confirm effectiveness, dosage, method of application, and specific action of individual polyphenolic components, as well as different extraction methodologies and solvents used, isolation and data processing [368,369,370]. Although the use of propolis has been widespread since ancient times as a folk remedy, and after the First and Second World Wars, its use as a natural antibiotic increased considerably, as well as research to determine its chemical and pharmacological properties. The quality of propolis is very different with regard to the chemical profile and relative biological activity, which depends on the plant species in a certain geographical area. The standardization of propolis is necessary for its clinical application. In addition to different components with regard to the geographical area, the propolis composition is affected by the time of collection, storage conditions, solvents for propolis, weather conditions, environmental pollution, and inclusion of polluting waxes, among others [5,6,7,8,9,371]. For now, its application in the clinic is limited despite numerous confirmed data in in vivo models. Many studies have confirmed that propolis (safe and well-tolerated) can be recommended as an adjuvant agent against various cancers as it might potentiate either standard chemotherapy or radiotherapy (palliative) while suppressing the adverse effects induced by the standard chemotherapeutic drugs. Attention should also be paid to the applied dose of propolis, especially in the combined use with chemotherapeutics where the use of lower doses is expected. In contrast, part of the research shows the positive effects of polyphenols and a clear relationship between the intake of a certain dose of polyphenols and their protective effect. One of the research problems is to distinguish which polyphenols are responsible for various positive effects because more than 10,000 polyphenolic molecules are known, and only some of the polyphenolic molecules have been well researched [371]. Another problem is the low solubility of propolis in water, from 7 to 12%. In ethyl alcohol, it dissolves from 50 to 75% or higher by heating, and in acetone from 20 to 40%. The solubility depends on the duration of the extraction, the temperature of the solvent (ethanol, water, polyethylene glycol or methanol, hydroalcoholic extracts, chloroform, ether, and acetone), and the size of the propolis particles (it is best if it is in powder form). Poor bioavailability due to low water solubility could be overcome by the application of particles (NP, carriers-Ag-silica/Au) or encapsulation [20,139,180,372,373,374,375,376,377,378,379,380,381], which could enhance the chemopreventive effect. These approaches include the preparation of gold nanoparticles, silver nanoparticles, magnetic nanoparticles, liposomes, liquid crystalline formulations, solid lipid nanoparticles, mesoporous silica nanoparticles, etc. NPs-encapsulated drugs are able to protect drugs from enzymatic degradation, control their release, prolong their blood level, change their pharmacokinetics, and decrease their toxicity via limiting non-specific uptake. It seems that further research into propolis and its bioactive constituents and their target molecules is needed for the safe use of propolis in humans, as clinical trials require standardized quality control and perfect drug design. Furthermore, the ratio of propolis compounds in humans should be investigated, as well as how to avoid allergic reactions to propolis.

Despite the many positive properties of propolis and its components, propolis, as well as some of its chemical ingredients, can cause an allergic reaction, the frequency of which varies between 1.2% and 6.55%. So far, as many as 26 allergens have been isolated, among which the strongest known sensitizers that cause allergic reactions such as caffeic acid esters (phenylethyl caffeate and methylbutenyl caffeate). Caffeic acid esters play an important role in propolis sensitization (contact dermatitis, oral mucositis with ulceration), and the responsible contact allergens are 3-methyl-2-butenyl caffeate and phenylethyl caffeate. Furthermore, Isoprenyl caffeate present in the composition of propolis is indicated as pro-hapten, which can be enzymatically oxidized in skin cells and present as an allergen for T-cells (see review papers [276]). In addition, the group of caffeic acid ester allergens also includes 1,1-dimethylallyl caffeic acid ester, benzyl caffeate, geranyl caffeate) and cinnamic acid esters, including cinnamyl cinnamate, benzyl cinnamate, and cinnamyl alcohol). The most important contact allergen identified in propolis is compound LB-1 (consisting mainly of three pentenyls caffeic acid esters) obtained from poplar buds. The GC/MS analysis determined the exact composition of LB-1. That consists of 3-methyl-2-butyl-caffeate (54.2%), 3-methyl-3- butyl caffeate (28.3%), 2-methyl-2-butyl caffeate (4.3%), phenethyl caffeate (7.9%), caffeic acid (1.3%) and benzyl caffeate (1.0%) [276]. Another important contact allergen in propolis is phenethyl ester of caffeic acid (phenethyl caffeate (CAPE)), which is present in smaller amounts than LB-1. After applying the same low concentrations (0.1%) these two allergens show a similar response. The third contact allergen is benzyl salicylate, which is a medium-strength allergen but can lead to cross-reactions in patients allergic to propolis. Benzyl cinnamate, as the fourth contact allergen, has weak allergenic properties. On the other hand, other authors suggest that propolis and its components are effective in controlling allergic diseases because many antioxidants inhibit the degranulation of mast cells and the release of histamine, tryptase, leukotriene, prostaglandin D2, and granulocyte and macrophage colony-stimulating factor (GM-CSF), IL-6, IL-8, and other mediators of inflammation. In addition, flavonoids are inhibitors of the production of histamine, IL-4, IL-5, and IL-13 when activated by human basophils. Flavonoids have the ability to downregulate the inflammatory chemokines IL-1β, IL-3, IL-4, IL-5, IL-9, IL-12 p40, IL-13, IL-17, TNF-α, G-CSF, GM-CSF, MCP-1, MIP-1α, MIP-1β, and RANTES by modulating the NF-κB pathway [5,276]. The mechanism of flavonoid inhibition of mast cell degranulation is a consequence of receptor-directed modulation of Ca^2+^ channels in the plasma membrane and/or calmodulin. A detailed description of anti-inflammatory, antioxidant, and anti-allergic mechanisms is described in the paper [276]. It seems that the anti-inflammatory effect of propolis is due to the presence of polyphenols/flavonoids (galangin, quercetin, naringin, especially galangin, acacetin, phenyl ester of caffeic acid (CAPE) and caffeic acid (CA)). These propolis components are responsible for the inhibition of COX and LOX activity, reduction of PGE2 release, nitric oxide (NO), and the expression of the inducible COX isoform (COX-2). In addition, propolis has an inhibitory effect on the activity of myeloperoxidase, NADPH oxidase, ornithine decarboxylase, tyrosine-protein-kinase, and hyaluronidase. Thus, propolis and its flavonoids effectively reduce inflammation, probably through the inhibition of NF-κB, a redox-sensitive transcription factor that controls a number of genes involved in inflammation, proliferation, cell survival, and apoptosis. NF-κB) signaling pathway has emerged as a critical player in the development of drug resistance in cancer cells. The process of drug resistance in tumors is intricately linked to the regulation of cell death and innate immunity. NF-KB affects numerous signaling pathways of cell death, including processes of regulation of apoptosis, autophagy, ferroptosis, pyroptosis, necroptosis, innate and adaptive immunity pathways, as well as other signaling pathways such as HIF-1. It is also involved in resistance to chemotherapy, radiotherapy, endocrine therapy, and immunotherapy. NF-κB has been shown to directly regulate PD-L1 expression in macrophages and other myeloid cells via inflammatory cytokines (IL-12, IFN-γ), PAMPs, and DAMPs.

## 7. Conclusions and Recommendations for Future Studies

Our data, along with findings from other researchers, demonstrate that propolis and its flavonoids can increase the sensitivity of tumor cells to chemotherapy and radiotherapy while simultaneously reducing unwanted side effects, particularly in cases of multidrug resistance and tumor recurrence. Additionally, they help protect healthy cells from the toxic effects of these therapies. The interaction of propolis and flavonoids with different cancer therapies leads to enhanced apoptosis, inhibition of angiogenesis, improved anti-cancer activity, reduced tumor invasion and metastasis, and suppression of resistance mechanisms while minimizing toxicity to multiple tissues and organs. The improved anti-cancer effects are largely attributed to the upregulation of apoptotic pathways and downregulation of NF-κB signaling. Propolis and its flavonoids target NF-κB by modifying the tumor microenvironment, inhibiting EMT, regulating growth factor receptors, and modulating key pathways such as PI3K/Akt, MAPK/ERK, and Janus kinase/signal transduction in cancer cells. Moreover, the modulation of NF-κB signaling by flavonoids affects ATP-binding cassette transporters, apoptosis, autophagy, cell cycle, and the activity of CSCs, oncogenes, and DNA repair mechanisms.

The reduction in side effects is linked to the activation of Nrf2-regulated antioxidant mechanisms. Propolis has demonstrated immunomodulatory properties by enhancing the activity and cytotoxicity of NK cells and the cytotoxicity of cytotoxic T-lymphocytes against tumor cells while downregulating regulatory T-cells (Tregs), which often protect tumors from immune attacks. Additionally, flavonoids exhibit anti-inflammatory effects by suppressing the release of pro-inflammatory cytokines, blocking the NF-κB and NLRP3 pathways, and reducing chemokine synthesis. Flavonoids can also target the tumor microenvironment by reprograming Tregs and TAMs, reducing angiogenesis, and decreasing the number of immunosuppressive cells or reactivating their anti-tumor functions. This includes reducing the expression of PD-1 and/or PD-L1 on T-cells.

Nanoparticles offer a promising solution for enhancing the therapeutic potential of flavonoids, either alone or in combination with other treatments. Nanomedicine, i.e., using lipid nanoparticles, protein nanoparticles, liposomes, metallic nanoparticles, and silica nanoparticles, has shown advantages over conventional therapies: (i) more precise drug delivery to targeted sites, (ii) better accumulation of drugs in the tumor tissues due to enhanced permeability and retention, and (iii) improved pharmacokinetic properties. Such approaches can enhance the antiangiogenic effects of therapies, block the suppressive effect of tumor microenvironment on immune cells, and directly increase the cytotoxic effect on tumor cells, ultimately improving therapeutic outcomes.

In summary, propolis and its polyphenolic/flavonoid components offer the potential to overcome drug resistance and enhance the efficacy of cancer therapies agents through several mechanisms: (i) increasing drug cytotoxicity and promoting apoptosis; (ii) overcoming drug resistance by inhibiting P-gp-mediated drug efflux; (iii) synergizing with chemotherapeutics targeting topoisomerase I and II; (iv) inhibiting protein tyrosine kinases; (v) downregulating NF-κB; (vi) modulating phase I and II detoxification enzymes; vii) stimulating the immune system; (viii) mimicking steroid hormones (phytoestrogen activity); (ix) inhibiting angiogenesis; (x) blocking mitotic signals; (xi) downregulating oncogenes and activating tumor suppressor genes; (xii) inhibiting pro-oxidative enzymes; (xii) regulating epigenetic enzymes like DNA methyltransferase, histone acetyltransferase, and histone deacetylase; and (xiii) differentiating between healthy and cancerous microenvironment. The interaction between propolis and chemotherapeutic agents may also lead to food–drug interactions, potentially altering the bioavailability of co-administered drugs.

Moreover, chemotherapy can enhance the effects of immunotherapy through mechanisms such as (i) inhibiting immunosuppression mediated by Tregs and myeloid-derived suppressor cells, (ii) promoting the maturation of dendritic cells and enhancing their ability to present tumor antigens, (iii) boosting the activity of cytotoxic T-cells, and (iv) increasing the infiltration of immune cells into the tumor core.

In conclusion, propolis and its polyphenolic/flavonoid components can complement conventional cancer therapies, improve treatment outcomes, and minimize undesirable clinical consequences.
